# MOB1 Mediated Phospho-recognition in the Core Mammalian Hippo Pathway * [Fn FN2]

**DOI:** 10.1074/mcp.M116.065490

**Published:** 2017-04-03

**Authors:** Amber L. Couzens, Shawn Xiong, James D. R. Knight, Daniel Y. Mao, Sebastian Guettler, Sarah Picaud, Igor Kurinov, Panagis Filippakopoulos, Frank Sicheri, Anne-Claude Gingras

**Affiliations:** From the ‡Lunenfeld-Tanenbaum Research Institute, Sinai Health System, Toronto, Ontario, Canada, M5G 1X5,; §Department of Biochemistry, University of Toronto, Toronto, Ontario, Canada, M5S 1A8;; ¶New address: The Institute of Cancer Research, Divisions of Structural Biology and Cancer Biology, London, UK, SW7 3RP;; ‖Structural Genomics Consortium, University of Oxford, Old Road Campus Research Building, Roosevelt Drive, Oxford OX3 7DQ, U.K.;; **NE-CAT APS, Building 436E, Argonne National Lab, 9700 S. Cass Avenue, Argonne, Illinois 60439;; ‡‡Ludwig Institute for Cancer Research, University of Oxford, Oxford OX3 7DQ, U.K;; §§Department of Molecular Genetics, University of Toronto, Toronto, Ontario, Canada, M5S 1A8

## Abstract

The Hippo tumor suppressor pathway regulates organ size and tissue homoeostasis in response to diverse signaling inputs. The core of the pathway consists of a short kinase cascade: MST1 and MST2 phosphorylate and activate LATS1 and LATS2, which in turn phosphorylate and inactivate key transcriptional coactivators, YAP1 and TAZ (gene WWTR1). The MOB1 adapter protein regulates both phosphorylation reactions firstly by concurrently binding to the upstream MST and downstream LATS kinases to enable the trans phosphorylation reaction, and secondly by allosterically activating the catalytic function of LATS1 and LATS2 to directly stimulate phosphorylation of YAP and TAZ. Studies of yeast Mob1 and human MOB1 revealed that the ability to recognize phosphopeptide sequences in their interactors, Nud1 and MST2 respectively, was critical to their roles in regulating the Mitotic Exit Network in yeast and the Hippo pathway in metazoans. However, the underlying rules of phosphopeptide recognition by human MOB1, the implications of binding specificity for Hippo pathway signaling, and the generality of phosphopeptide binding function to other human MOB family members remained elusive.

Employing proteomics, peptide arrays and biochemical analyses, we systematically examine the phosphopeptide binding specificity of MOB1 and find it to be highly complementary to the substrate phosphorylation specificity of MST1 and MST2. We demonstrate that autophosphorylation of MST1 and MST2 on several threonine residues provides multiple MOB1 binding sites with varying binding affinities which in turn contribute to a redundancy of MST1-MOB1 protein interactions in cells. The crystal structures of MOB1A in complex with two favored phosphopeptide sites in MST1 allow for a full description of the MOB1A phosphopeptide-binding consensus. Lastly, we show that the phosphopeptide binding properties of MOB1A are conserved in all but one of the seven MOB family members in humans, thus providing a starting point for uncovering their elusive cellular functions.

First identified in *Drosophila melanogaster*, the Hippo signaling pathway controls organ size through the regulation of cell proliferation, apoptosis, and contact inhibition (1–7). The pathway is molecularly and functionally conserved from flies to mammals; however, in mammals, paralogous proteins with apparent redundant functions exist for several of the pathway members. Consistent with a tumor suppressor function, dysregulation of the Hippo pathway is associated with human cancers (reviewed in 8, 9) though the core components themselves are infrequently mutated (10–12), possibly because of a redundancy of function arising from the presence of paralogous proteins.

The canonical mammalian Hippo pathway consists of a short kinase cascade in which mammalian STE20-like protein kinase 1 and 2 (MST1 and MST2 kinases) ^1^ (gene names *STK4* and *STK3;* Hpo in flies) phosphorylate and activate the downstream kinases LATS1 and LATS2 (Warts in flies 6, 13). Activated LATS1 and LATS2 then phosphorylate the transcriptional coactivator YAP1 and TAZ (gene names *YAP1* and *WWTR1*; Yorkie in flies), leading to their cytoplasmic sequestration and proteasome-mediated degradation (14). The core kinase cascade is modulated by adaptor proteins, SAV1 (Salvador, also known as WW45) and MOB1 (encoded by *MOB1A* and *MOB1B* in human), both of which work to promote Hippo pathway signaling.

MOB1 directly binds to LATS1 and LATS2, and additionally to two related kinases, NDR1, (gene name *STK38*) and NDR2 (gene name *STK38L*) via a specialized MOB1-binding domain N-terminal to the kinase domain (15; also see accompanying manuscript by Xiong *et al.*, 2017). This binding interaction potentiates their kinase activity both *in vitro* and *in vivo* (16–19). In addition to its role as a direct activator of the NDR and LATS kinases, MOB1 itself is a substrate of MST1 and MST2, and this phosphorylation increases the ability of MOB1 to activate LATS1 and LATS2 (20, 21).

Key to its function as a Hippo pathway activator is the ability of MOB1 to bind directly to MST1 and MST2 kinases. Recent structural studies provided key insight into the underlying mechanism of binding. Rock *et al.* first demonstrated that yeast Mob1 can bind in a phosphorylation-dependent manner to the Mitotic Exit Network regulator Nud1 (22). Mutational analysis identified a short linear phosphopeptide motif within Nud1 as being sufficient for high-affinity interaction (*K_d_* = 2.4 μm for peptide sequence TVLNNYSpTVHQKVPS). Optimization with peptide library arrays further demonstrated that yeast Mob1 displayed a preference for pSer over pThr at the P0 position. The basis for phosphopeptide recognition and its conservation in human MOB1 was then demonstrated by a crystal structure of human MOB1 bound to an optimized Nud1-like phosphopeptide. The structure revealed an infrastructure for binding phosphate moieties involving three basic residues (K153, R154 and R157 in human MOB1, 22).

Subsequent studies by our group first demonstrated a link between the phospho-recognition abilities of MOB1 and mammalian Hippo pathway signaling. Couzens *et al.* performed interaction proteomics studies revealing that MOB1 recruits MST1 and MST2 in a phosphorylation-dependent manner (23) using the conserved phospho-recognition infrastructure involving K153, R154, and R157 first discovered by Rock *et al.* (22).

Ni *et al.* followed up on these findings by solving the crystal structure of human MOB1B in complex with a phosphothreonine-containing peptide sequence corresponding to residues 371 to 400 of MST2. In addition to confirming the generality of the phospho-recognition mechanism predicted from prior studies (22, 23), the structure revealed a bipartite binding mode, in which the phosphorylated T378 moiety of MST2 bound the phospho-binding pocket of MOB1B, whereas a hydrophobic motif encompassing residues 390 to 398 of MST2 additionally engaged a remote secondary surface on MOB1B.

Open questions of MOB1 function that remain to be addressed include first, what is the precise sequence preference of human MOB1 for phosphosites in target interactors? The MOB1 interacting sequences in human MST2 and in yeast Nud1 employ pThr at the P0 position, however, these pThr peptides are suboptimal when compared with the preferred choice of pSer peptides demonstrated for yeast Mob1 in a peptide library screen (22). Second, how does the phosphopeptide-binding consensus of MOB1 compare with the substrate phosphorylation consensus of the upstream MST1 and MST2 targeting kinases? Do the similarities and differences in consensus preferences account for the specificity of sites generated and engaged by MOB1 in a biological setting? Thirdly, is the phosphopeptide binding function of MOB1 a general feature of the other six human MOB proteins?

To address these questions, we employed phosphoproteomics, seeking to characterize the sequence determinants of substrate phosphorylation by MST1 and MST2. Using peptide arrays, quantitative binding analyses, and structure determinations, we also characterized the sequence dependences required for high-affinity phosphopeptide binding to MOB1. Our results help explain the observed redundancy in a cellular context of several phosphorylation sites on MST1 (including the bipartite site crystallized by Ni *et al.*, 21). Furthermore, we demonstrate that all but one of the human MOB proteins share the ability to bind a MST1 phosphopeptide, thus providing a foundation for future investigations to elucidate the function of other MOB family members that have poorly understood biological functions.

## EXPERIMENTAL PROCEDURES

### 

#### 

##### Recombinant Protein Expression and Purification

Human MST1 kinase domain (amino acids 2–326, corresponding to NCBI RefSeq protein NP_006273), MST2 kinase domain (2–322, NP_006272), MOB1B (2–216, NP_001231695), MOB1A (2–216, NP_060691) wild-type and indicated MOB1A mutant proteins, MOB2 (2–237, NP_001165694), MOB3A (2–217, NP_570719), MOB3B (2–216, NP_079037), MOB3C (2–216, NP_660322), MOB4 (2–225, NP_056202), MOB4^RRR→AAA^ (2–225), and yeast Mob1 (79–314, NP_012160) were expressed in *E. coli* BL21 (DE3) CodonPlus RIL cells as N-terminal dual 6xhistidine (HIS) and glutathione S-transferase (GST), Tobacco Etch Virus (TEV) cleavable, fusion proteins using a modified pETM-30 vector. MOB wild-type and mutants were purified in batch on glutathione-Sepharose resin (GE Healthcare, Uppsala, Sweden) and eluted by cleavage from the affinity tags with HIS tagged TEV protease. TEV protease was removed from the eluted protein by subtractive immobilized-metal affinity chromatography. Cleaved protein was then concentrated and buffer exchanged by size exclusion chromatography (SEC) using a Superdex 75 120 ml column (GE Healthcare). For GST pull-downs and Far-Western analysis, the TEV cleavage step was omitted.

##### Peptides for Biophysical and Structural Studies

MST1 phosphopeptides encompassing pT353 and pT367 (15-mers) used for crystallization and FITC labeled nonphosphorylated and phosphorylated MST1 T353 peptides, T367 peptides, T329-T340 peptides, T380-T387 peptides and optimized Nud1-like peptides for fluorescence polarization binding experiments were purchased from Biomatik (Cambridge, ON). Peptide sequences used for crystallization studies are: *T353, VASTMTDGAN(p)TMIEH and T367, DDTLPSQLG(p)TMVINA.* All peptides used for biophysical studies are listed in supplemental Table S1.

##### Crystallization and Data Collection

Complexes of MOB1A with MST1 phosphopeptides (pT353 and pT367) were obtained by mixing at a 1:1.5 mole ratio. Crystals of the MOB1A - MST peptide complexes were obtained by vapor diffusion using hanging drops with 1:1 mixtures of protein (at final concentration of 7 mg/ml) with a precipitant solution of 0.1 m MES pH 6.0, 0.2 m LiCl and 20% PEG 6000. For diffraction studies, all protein crystals were flash-frozen in mother liquors supplemented with 20–25% (v/v) ethylene glycol.

Crystals of MOB1A+pT353 peptide complex and MOB1A+pT367 peptide complex with the common space group P4_3_2_1_2 (a = 60.986, b = 60.986, c = 138.486, alpha = 90, beta = 90, gamma = 90) contained one molecule per asymmetric unit. Diffraction data collection was performed at the NE-CAT beamline 24-ID-E (Advanced Photon Source, Argonne National Laboratory, Argonne, IL).

##### Structure Solution and Refinement

X-ray data sets were processed using the HKL2000 software (24). MOB1A+MST1 phosphopeptide structures were solved by molecular replacement using the MOB1A core domain (PDB ID: 1PI1, 25) as a search model. Model autobuilding was carried out in Buccaneer to build the N-terminal extension of MOB1A (26). Model manual building was conducted in COOT (27) and refined with Refmac (28). Clear unbiased density allowed unambiguous modeling of the ordered regions of the MST1 phosphopeptides. See supplemental Table S1 for data collection and model refinement statistics. X-ray costructures of MOB1A+pT353 and MOB1A+pT367 have been deposited to the Protein Data Bank with PDB codes 5TWH and 5TWG, respectively.

##### Fluorescence Polarization Peptide Binding Assay

Twelve-point fluorescence polarization binding measurements were performed in triplicate in 384-well plate format using an Analyst HT (Artisan-Scientific, Sunnyvale, CA) reader. Each 20 μl measurement condition contained 20 nm FITC labeled MST1 peptide or Nud1-like peptide and the indicated concentration of MOB proteins in 25 mm HEPES pH 7.5, 150 mm NaCl, 2 mm DTT, and 1 mg/ml bovine serum albumin (BSA). *K_d_* values were obtained by fitting the results to a one site-specific binding model using GraphPad Prism.

##### Peptide Arrays and Far-Western blots

Cellulose-bound peptide arrays were prepared employing Fmoc solid phase peptide synthesis using a MultiPep-RSi-Spotter (INTAVIS, Köln, Germany) according to the SPOT synthesis method provided by the manufacturer, and previously described (29). Peptides were synthesized on amino-functionalized cellulose membranes (Whatman™ Chromatography paper Grade 1CHR, GE Healthcare Life Sciences #3001–878) and the presence of SPOTed peptides was confirmed by ultraviolet light (UV, λ = 280 nm). Peptide sequences were chosen for each of the phosphorylated sites within the sequence encompassing residues 327 to 487 of MST1 as reported in PhosphositePlus (31/01/2014, ref 30). In addition, 15 amino acid-long peptides spanning the entire MST1 linker region (aa 327–487) were also synthesized in both phospho- and nonphosphorylated versions (for every serine, threonine, or tyrosine position; supplemental Fig. S1). Subsequent arrays were generated by alanine scanning from the pT353 and pT367 peptide sequences. The peptide arrays were soaked in 100% ethanol and blocked in 5% milk in H_2_O for 6 h at 20 °C. One microgram of GST-MOB1B (or GST alone) was used to probe the arrays at 20 °C overnight. Following washes with PBS plus 0.1% Tween-20, the arrays were blocked again in 5% milk for 1 h. The arrays were next probed with anti-GST coupled to horseradish peroxidase for 1 h and imaged with LumiGLO (Cell Signaling Technology, Danvers, MA) on film.

For Far-Western blots, samples were first resolved by 10% SDS-polyacrylamide gel electrophoresis and transferred to a nitrocellulose membrane. Membranes were then incubated with 1 μg of GST-MOB1B and processed as described for the peptide arrays (except that the soaking in ethanol was omitted).

##### Western Blots

Samples were resolved on 10% SDS-polyacrylamide gel electrophoresis and transferred onto a nitrocellulose membrane, probed with the primary antibodies as indicated in legends, followed by fluorescent secondary antibodies for mouse (CW800, LICOR) or rabbit (CW680, LICOR, Lincoln, NE), and then directly imaged on an Odyssey scanner (LI-COR). M2-FLAG antibody was from Sigma-Aldrich (F1804), and anti-GST Horseradish Peroxidase (HRP) was from GE Healthcare (RPN1236).

##### Mammalian Cell Expression

BirA*-FLAG or 3xFLAG tagged MST1 constructs were generated via Gateway cloning into pDEST5 BirA FLAG pcDNA5 FRT TO or pDEST 5′ Triple FLAG pcDNA5 FRT TO, respectively (23). The accession number for the starting clone was BC093768. Point mutations were generated by polymerase chain reaction-directed mutagenesis (the position of the mutated amino acids is indicated based on the reference sequence). All constructs were sequence-verified. Stable cell lines were generated as Flp-In 293 (or HeLa) T-REx cell pools for the BirA*-FLAG-MST1 constructs as described (31), and expression was induced for 24 h with tetracycline (1 μg/ml). 250 ng of 3xFLAG-MST1 constructs were transiently transfected into HEK293 cells in 6-well format with jetPRIME (Polypus transfection, Illkirch, France) as per the manufacturers' instructions. Okadaic acid was added at a concentration of 150 nm for 2.5 h unless otherwise indicated, and DMSO was used as a negative control.

##### GST Pull-down Coupled to Western blot

To check the binding of the MST1 proteins expressed from a mammalian system to MOB1B by GST pull-down assays, cell expressing the indicated FLAG or BirA*-FLAG-tagged MST1 constructs were lysed in CHAPS lysis buffer [40 mm Hepes (pH 7.5), 120 mm NaCl, 1 mm EDTA, 0.3% CHAPS] with 1× protease inhibitors (Sigma-Aldrich, St. Louis, MO) after treatment with 150 nm okadaic acid for 2.5 h. Lysate was then incubated for 2 h with ∼ 1 μg GST-MOB1 immobilized onto 20 μl packed glutathione-Sepharose beads. Beads were washed 3 times with CHAPS buffer, and SDS sample buffer was added directly to the beads for elution and loading onto SDS-PAGE gels which were processed for immunoblotting as described above.

##### Determination of the MST1 and MST2 Phosphorylation Consensus

To determine phosphorylation consensus for recombinantly expressed MST1 and MST2 kinase domains, a whole-cell lysate kinase assay was employed (32). Essentially: HeLa cells were grown to confluency in SILAC (33) media (DMEM, 10% dialyzed FBS, 1% penicillin/streptomycin + 50 mg/L of light (K0, R0), medium (K4, R4) or heavy (K8, R10) amino acids). Cells were pelleted in ice-cold PBS. Frozen pellets were thawed into Nonidet P-40 buffer (50 mm Tris-HCl, pH 7.5, 150 mm NaCl, 1% Nonidet P-40, 1 mm PMSF, 1:500 Sigma-Aldrich protease inhibitor mixture P8340), at 500 μl per plate of cells. Two hundred fifty units of benzonase (Novagen, Billerica, MA) were added per 2.5 ml of lysis buffer and the sample was passed through a 25 gauge needle five times. The sample was then sonicated for 30 s (10 s on, 2 s off) and then placed at 4 °C for 30 min with rotation. The sample was then spun at 10,000 × *g* for 10 min to pellet debris. Protein concentration was determined by Bradford assay (Bio-Rad, Mississauga, ON). 3 mg aliquots of SILAC-labeled lysate were treated with 100 μl (packed) Phos-Select IMAC resin (Sigma-Aldrich) for 1 h at 4 °C to remove abundant and heavily phosphorylated proteins (resin was prewashed twice with mass spectrometry-grade water). The resin was removed by centrifugation and the supernatant collected. The lysate was treated with 5′-(4-fluorosulfonylbenzoyl)adenosine (FSBA) at a concentration of 20 mm (in DMSO) for 1 h at 30 °C at a protein concentration of 2 mg/ml to inhibit all endogenous kinases. Following FSBA-treatment, 3 mg lysate aliquots were diluted with 4.35 ml of desalting buffer (50 mm Tris-HCl, pH 7.5, 150 mm NaCl) and precipitated FSBA was removed by centrifugation at 10,000 × *g*. Samples were then desalted and concentrated to 4 mg/ml using Pierce 7 ml 9kDa molecular weight cutoff concentrators (catalogue: 87748). The lysate was then diluted with one volume of 2x kinase assay buffer (40 mm MOPS, pH 7.2, 50 mm β-glycerophosphate, 10 mm EGTA, 2 mm NaVO_4_, 2 mm DTT, 20 mm MgCl_2_, 20 mm MnCl_2_, 2 mm ATP).

Recombinant MST1 and MST2 were then added to the inactivated lysate at 1 μg/mg of lysate to the medium and heavy SILAC samples, respectively (swapped for replicates), and whole lysate kinase reactions were performed at 30 °C for 2 h. For the reduction of cysteines, DTT was added to 5 mm and the samples placed at 60 °C for 15 min, then allowed to cool for 15 min at room temperature. For alkylation, iodoacetamide was added to a final concentration of 10 mm and the sample left for 30 min at room temperature in the dark. The SILAC samples (L, M, H) were then pooled and CaCl_2_ added to 10 mm. The sample was split in half and each half was digested with either trypsin or chymotrypsin at a ratio of 1:60 w/w overnight at 37 °C. In the morning, an additional 40 μg of trypsin or chymotrypsin was added, and the digestion incubated for another 3 h. The sample was acidified to pH 2 with formic acid and desalted using 200 mg of Sep-Pak C18 (37–55 μm particle size, Waters, catalog: 186004618) in a vacuum manifold column. The desalted sample was vacuum centrifuged to 100 μl and diluted with 300 μl of RBD buffer (35% lactic acid, 60% ACN, 2.5% TFA). 40 mg of TiO_2_ was washed thrice, sequentially with methanol, H_2_O and LAS (25% lactic acid, 60% ACN, 2.5% TFA) and then divided into two 20 mg aliquots. One aliquot was added to the lysate and left to nutate for 15 min. Unbound supernatant was then added to the other TiO_2_ aliquot suspend in DB (60% ACN, 2.5% TFA) for 15 min. Following incubation, the TiO_2_ aliquots were pooled and washed sequentially in LWS (12.5% lactic acid, 60% ACN, 2.5% TFA), 80% ACN + 0.1% TFA, 0.1% TFA and H_2_O. Phosphopeptides were sequentially eluted with 200 μl of 50 mm ammonium bicarbonate, 5% NH_4_OH, 5% piperidine, 5% pyrrolidine and each eluted fraction acidified with formic acid to pH 2.7. Finally, the samples were desalted using 75 μl of packed Poros 20 R2 resin in a microspin column. The piperidine and pyrrolidine fractions were pooled for analysis.

Samples were analyzed by nano-LCMS using a home-packed 0.75 μm x 10 cm C18 emitter tip (Reprosil-Pur 120 C18-AQ, 3 μm). A NanoLC-Ultra HPLC system (Eksigent, Dublin, CA) was coupled to an LTQ Orbitrap Velos (Thermo Fisher Scientific, Waltham, MA) and samples were analyzed in data-dependent acquisition mode. High-resolution MS scans (60,000) were followed by data dependent selection of the top 15 ions for MS/MS in CID mode with 20 s exclusion times. The LC gradient was delivered at 200 nl/m and consisted of a ramp of 2–35% acetonitrile (0.1% formic acid) over 90 m, 35–80% acetonitrile (0.1% formic acid) over 5 ms, 80% acetonitrile (0.1% formic acid) for 5 m, and then 2% acetonitrile for 20 m. The raw files and Maxquant output folder that can be used for visualization of the results (see below) are deposited in the MassIVE repository housed at the Center for Computational Mass Spectrometry at University of California, San Diego (UCSD) (http://massive.ucsd.edu). This partial MassIVE submission has been assigned the MassIVE ID MSV000080315, ftp://massive.ucsd.edu/MSV000080315.

##### Mass Spectrometric Data Analysis for the MST1 and MST2 Kinase Consensus

Raw files were searched and quantified using Maxquant (34) version 1.2.2.5 using the Uniprot database (84887 sequences, 20 November, 2012). Cysteine residues were searched as a fixed modification of +57.0215 Da, oxidized methionine residues as a variable modification of +15.9949 Da and deamidated asparagine and glutamine residues as a variable modification of +0.9840. Medium and heavy SILAC labeling of lysine (K) and arginine (R) residues were set as variable modifications of +4 Da for medium R and K and +10Da for heavy R and +8Da for heavy K. Peptides were queried using either trypsin or chymotrypsin cleavage constraints with a maximum of two missed cleavages sites. The mass tolerances were 6 ppm for precursors masses and 0.5 Da for fragment masses. The peptide false-discovery rate was set to 0.01. Phosphopeptides with a SILAC ratio of at least 3 relative to the control in both replicates were accepted as target substrate peptides. The consensus phosphorylation motif was defined using amino acids ± 6 residues relative to the target site. Because 82% of sequences contained a target threonine, we used only these sequences in generating the consensus motif. The sequence logo was created using pLogo (35) with a background of all human protein sequences.

##### Determination of MOB4 Interactors by GST Pull-down Assays from Cellular Lysate

HeLa cells were grown to confluency and treated with 150 mm okadaic acid for 2.5 h. Cells were harvested in PBS and the pellet flash-frozen. The pellet was thawed in lysis buffer (50 mm Tris-HCl, pH 7.4, 100 mm NaCl, 10 mm MgCl_2_, 1% Nonidet P-40, 10% glycerol, 1 mm PMSF, 1 mm sodium orthovanadate, 100 mm NaF and 1:500 Sigma-Aldrich protease inhibitor mixture P8340) at a 1:4 ratio (pellet weight/volume) and resuspended by pipetting up and down. Two hundred fifty units of benzonase (Novagen) were then added and the sample sonicated for 30 s at amplitude 0.37 (10 s pulse, 2 s off). Following sonication, the sample was nutated at 4 °C for 30 min and then centrifuged at 18,000 × *g* for 20 min at 4 °C. The supernatant was passed through a 0.2 μm filter to remove any remaining cell debris. Twenty micrograms of packed glutathione beads were washed twice in PBS, and incubated with either PBS or bacterial lysate containing either GST-tagged MOB4 WT or MOB4^RRR→AAA^ mutant for 30 min and subsequently washed three times with PBS. Two milligrams of centrifuged cell lysate was added to the glutathione beads and incubated for 2 h at 4 °C with gentle agitation. The beads were then pelleted by centrifugation at 1000 × *g* for 1 min. The supernatant was removed and the beads washed sequentially once with 500 μl of cold lysis buffer and twice with 500 μl of cold wash buffer (50 mm Hepes-NaOH pH 8.0, 150 mm NaCl, 2 mm EDTA, 0.1% Nonidet P-40 and 10% glycerol). The beads were transferred to a fresh microcentrifuge tube and washed twice with 500 μl of 20 mm Tris-HCl, pH 8. Proteins were eluted from the beads using 0.5 m NH_4_OH and the eluate was vacuum-centrifuged to dryness. Dried protein was resuspended in 100 μl of 50 mm ammonium bicarbonate, pH 8.5, containing 500 ng of trypsin and incubated at 37 °C overnight (∼15 h). In the morning, another 500 ng of trypsin was added in 20 μl of ammonium bicarbonate and the sample left for another 4 h. The digestion was terminated by the addition of 2% formic acid, the sample was then vacuum centrifuged to dryness.

##### Mass Spectrometric Analysis of GST Pull-downs

Peptides were analyzed as described above for the determination of the MST1 and MST2 consensus, except each 60,000 resolution MS scan was followed by 10 CID MS/MS ion trap scans. This data set consisting of 6 raw files and associated peak list and results files has been deposited in ProteomeXchange through partner MassIVE as a complete submission and assigned the MassIVE ID MSV000080566 and PXD005963, ftp://massive.ucsd.edu/MSV000080566.

##### Mass Spectrometry Data Analysis of GST Pull-downs

Raw files were converted to mzXML and mgf files using ProteoWizard 3.0.4468 (36) and analyzed using the iProphet pipeline (37) implemented within ProHits (38) as follows. The database consisted of the human and adenovirus sequences in the RefSeq protein database (version 57) supplemented with “common contaminants” from the Max Planck Institute (http://141.61.102.106:8080/share.cgi?ssid=0f2gfuB) and the Global Proteome Machine (GPM; http://www.thegpm.org/crap/index.html). The search database consisted of forward and reverse sequences (labeled “gi 9999” or “DECOY”); in total, 72,226 entries were searched. Spectra were analyzed separately using Mascot (2.3.02; Matrix Science) and Comet [2012.01 rev.3 (39)] with trypsin specificity and up to two missed cleavages; deamidation (Asn or Gln) and oxidation (Met) were selected as variable modifications. The mass tolerance of the precursor ion was set at ±12 parts per million (ppm), the fragment ion tolerance at ± 0.6 amu. The resulting Comet and Mascot results were individually processed by PeptideProphet (40) and combined into a final iProphet output using the Trans-Proteomic Pipeline (TPP; Linux version, v0.0 Development trunk rev 0, Build 201303061711). TPP options were as follows: general options were -p0.05 -x20 -d“gi 9999,” iProphet options were -ipPRIME, and PeptideProphet options were -OpdP. All proteins with a minimal iProphet probability of 0.05 were parsed to the relational module of ProHits. For analysis with SAINT, only proteins with an iProphet protein probability of >0.95 were considered. Hits were also restricted to those detected with a minimum of two unique peptides.

##### Interaction Scoring for the GST Pull-downs

Pull-downs were performed in duplicates and analyzed with SAINTexpress (41). Proteins with FDR of ≤1% and at least two unique peptides were considered true positive interactions. Visualization of the interactions as dot plots was through prohits-viz.lunenfeld.ca, which was first introduced in (42); once a particular prey passes the selected FDR threshold for at least one bait, all the quantitative data across all baits are retrieved and displayed. On these dot plots, the color intensity maps to the averaged spectral counts across both replicates (capped at the indicated maximal value), whereas the size of the circles indicates the relative abundance of a prey with regards to its maximum value. The confidence score (FDR) from SAINTexpress is mapped as the edge color.

##### Experimental Design and Statistical Rationale

For the GST pull-down data, biological duplicates were employed, and statistical scoring against two negative controls was performed using Significance Analysis of INTeractome (SAINT) as described in “Interaction Scoring for AP-MS”. Average SAINT score was used to determine the Bayesian FDR, which therefore requires a high confidence interaction across both biological replicates. For the consensus phosphorylation motif screen, technical replicates were performed for the control, MST1 and MST2 labeling experiments, permutating the SILAC labels between replicates as described above.

## RESULTS

### 

#### 

##### MOB1 Phosphorylation-dependent Interaction with MST1 is Direct and Maps to Regions C-terminal to the Kinase Domain

We previously demonstrated that MOB1 interacted in a phosphorylation-dependent manner with MST1, and that its association with MST1 and MST2 was abrogated by mutation of the MOB1B phosphate coordinating residues K153, R154 and R157. This suggested an underlying binding mode between MOB1 and MST1 like that revealed for MOB1 binding to a Nud1 derived phosphopeptide (22). However, inspection of the amino acid sequence of MST1 did not reveal obvious homology to the Nud1 derived phosphopeptide sequence (supplemental Fig. S1*A*). Because we surmised that the phospho-dependent binding of MST1 and MST2 to MOB1 could have functional consequences on Hippo pathway signaling and regulation, we set out to investigate the determinants for the MST1-MOB1 interaction in greater detail.

**Fig. 1.**
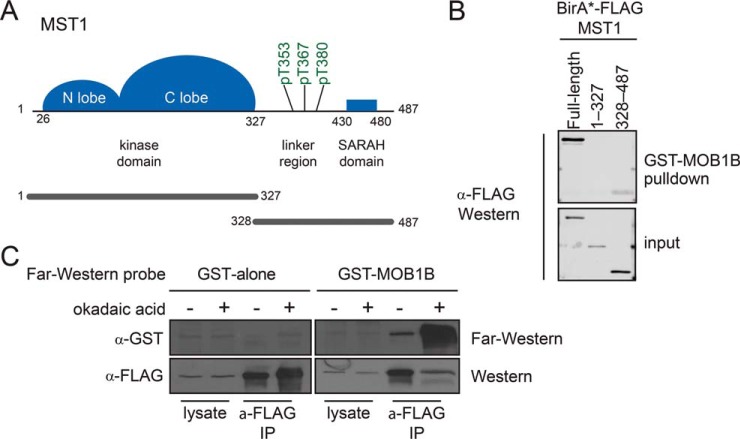
**Phospho-dependent interaction of MST1 kinase with MOB1.** *A,* MST1 kinase domain architecture, highlighting multiple phosphothreonine sites available for phospho-dependent interaction with MOB1. Constructs used for studies in panel B are highlighted. *B*, HeLa stable cell lines expressing full-length BirA*-FLAG-MST1, MST1 aa 1–327, and MST1 aa 328–487 were subjected to a GST-MOB1B pull down (top panel). Expression level controls for BirA*-FLAG-MST1 constructs are shown in the bottom panel. *C,* Far-Western analysis to study phosphorylation-dependent interactions with GST-MOB1B. HeLa cells stably expressing BirA*-FLAG-MST1 aa 328–487 at were treated with okadaic acid or vehicle (DMSO). BirA*-FLAG-MST1 was affinity purified, separated by SDS-PAGE, transferred to a nitrocellulose membrane and incubated with GST-MOB1B or with GST alone as a control. Signal was detected using anti-GST coupled to Horseradish Peroxidase and imaged with LumiGLO substrate on film. Control detection of immunoprecipitated FLAG-MST1 was performed by anti-FLAG Western blot.

MST1 is comprised of two structured domains: a protein kinase domain located at its amino terminus (aa 26–327; Fig. 1*A*) and a SARAH domain that mediates homo and hetero-dimerization at its carboxyl terminus (aa 430–480, ref 43). An unstructured linker region that harbors multiple phosphorylation sites (aa 328–429, ref 30, 44) separates the two structured domains. To identify the portion of MST1 necessary for recruitment to MOB1A, we stably expressed BirA*-FLAG-tagged full-length MST1, the kinase domain only (aa 1–327), and a C-terminal fragment containing both the linker and the SARAH domain (aa 328–487) in HeLa cells. Cells were treated with the protein phosphatase inhibitor okadaic acid to maximize the accumulation of phosphorylated proteins (23). Lysates were generated and subjected to pull-downs using recombinant bacterially-expressed GST-MOB1B immobilized on glutathione Sepharose resin. The recovery of MST1 was then monitored by immunoblotting using an anti-FLAG antibody (Fig. 1*B*). Full-length MST1 and the C-terminal fragment, but not the kinase domain alone, were recovered in the pull-down. We concluded that the MOB1B-binding region localized to the C-terminal portion of MST1.

To confirm that the interaction was phosphorylation-dependent, and to assess whether it was direct, we employed a Far-Western assay (45). We treated stable HeLa cells expressing BirA*-FLAG-MST1 C terminus (aa 328–487) with either DMSO (vehicle control) or okadaic acid. MST1 was recovered by anti-FLAG immunoprecipitation (to enrich for the expressed proteins and to improve assay sensitivity) and separated by SDS-PAGE. Proteins were transferred onto nitrocellulose, and Far-Western analysis was performed using GST alone or GST-MOB1B as probes (Fig. 1*C*; immunoblotting with anti-FLAG antibody was used to monitor relative expression levels and immunopurification efficiency, bottom blot). Although no binding signal was detected when GST-alone was used as probe, a band corresponding to MST1 was clearly visible in the FLAG-MST1 immunoprecipitates probed with GST-MOB1B. In addition, the intensity of the binding signal was increased severalfold in the okadaic acid-treated sample, confirming the expected phospho-dependence. These results demonstrated that the association between MST1 and MOB1 was direct and that the phosphorylation-dependent elements resided within MST1 itself (aa 328–487).

##### Phosphorylated T353 and T367 Containing 15-mer Peptides Within the Interdomain Linker of MST1 are Sufficient for Direct Binding to MOB1

We next repurposed the Far-Western assay with GST-MOB1B to identify the specific binding sites within the C terminus of MST1 using a peptide array strategy. We synthesized 15 amino acid-long peptides with the MST1 phosphorylation sites reported in PhosphositePlus between aa 328–487 (30), including both phosphorylated and nonphosphorylated versions of each. We further synthesized nonoverlapping 15-mers spanning the entire MST1 C-terminal region (aa 328–487) with combinatorial multisite phosphorylation when applicable. The phosphorylated and nonphosphorylated peptides derived from Nud1 (22) were used as positive and negative MOB1B binding controls, respectively. Peptide overlay analysis (here referred to as Far-Western) revealed that peptides bearing phosphorylated forms of T353 and T367 consistently gave the highest binding signals across the array; nonphosphorylated versions of these peptides generated little to no binding signal (supplemental Fig. S1*B*, S1*C*). Several phosphopeptides yielded intermediate binding signals, as did unexpectedly two nonphosphorylated peptides (supplemental Fig. S1*C*; discussed below).

**Fig. 2.**
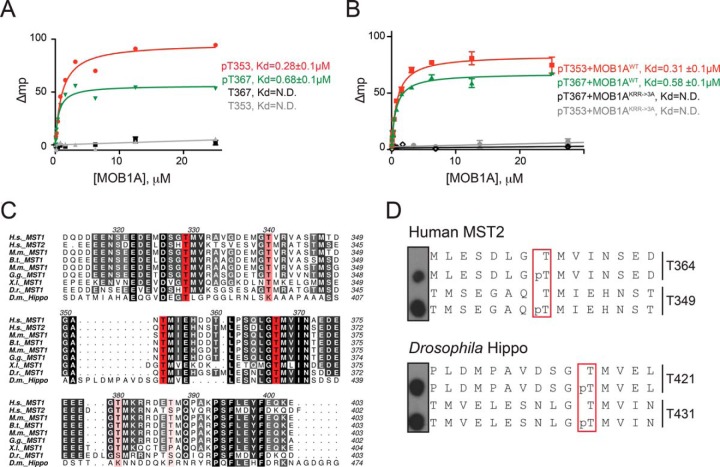
**Phosphopeptides from MST1 interdomain linker region directly interact with MOB1A *in vitro*.** *A–B,* Fluorescence Polarization measurement of the binding of MOB1A to phosphorylated and nonphosphorylated variants of MST1 T353 and T367 peptides (left). Fluorescent polarization measurement of binding between MOB1A WT or a KRR→3A mutant and the indicated MST1 phosphopeptides (right). Data is plotted as the mean ± S.E. (*n* = 3); N.D. indicates that the *K_d_* could not be determined. *C,* Sequence alignment of the inter domain linker region of MST1 and MST2 from humans to flies. The most conserved Thr residues are highlighted in red and less conserved Thr residues are highlighted in pink. H.s., *Homo sapiens*, M.m., *Mus musculus*; B.t., *Bos taurus*; G.g., *Gallus gallus*; X.l., *Xenopus laevis*; D.r., *Danio rerio*; D.m., *Drosophila melanogaster. D,* Peptide array studies to define MOB1B phospho-dependent interaction sites in human MST2 and *Drosophila* Hpo proteins that are orthologous to the human MST1 T353 and T367 sites.

To confirm that nonimmobilized peptides bearing phosphorylated T353 and T367 could bind MOB1A in solution, we conducted fluorescence polarization experiments using recombinantly-expressed wild-type MOB1A and fluorescently-labeled MST1 peptides. MST1 T353 and T367 peptides displayed high phospho-dependent binding affinities for MOB1A, with Kds of 280 nm and 680 nm for the phosphorylated peptides, respectively, and no detectable binding for the nonphosphorylated peptides (Fig. 2*A*). Consistent with our previous results indicating that coordination of the phosphate group by three basic amino acids in MOB1 (first identified in ref 22) was responsible for phosphorylation-induced interaction with MST1 (23), a recombinant MOB1A mutant bearing alanine substitutions at positions K153, R154, and R157 displayed no measurable binding to either of the phosphorylated peptides (Fig. 2*B*).

Like MST1, MST2 was also recruited to MOB1 in a phosphorylation-dependent manner (23), suggesting that the sequence elements that mediate MOB1 recruitment are conserved between MST1 and MST2. MST1 and MST2 share 59% identity in their C-terminal segments, and both T353 and T367 sites are conserved (Fig. 2*C*). We next validated that the paralogous sequences derived from MST2 also associated with MOB1B in a phosphorylation-dependent manner (Fig. 2*D*). We further noticed that the T353 and T367 sites are conserved in Hpo, the *Drosophila* MST1 and MST2 ortholog, though the sequence surrounding the site orthologous to T353 was somewhat divergent (Fig. 2*C*). Fly peptides orthologous to T353 and T367 also bound to human MOB1B in our Far-Western overlay assay in a phosphorylation-dependent manner (Fig. 2*D*; note that these binding experiments were performed with human MOB1B as we could not readily express the recombinant *Drosophila* MOB1 protein Mats in bacteria). These results suggested that the two conserved phosphorylation sites in MST1 are capable of mediating phosphorylation-dependent interactions with MOB1 across species.

##### The Phosphorylation Consensus of MST1 is a Close Match to the Phosphopeptide-binding Consensus of MOB1

**Fig. 3.**
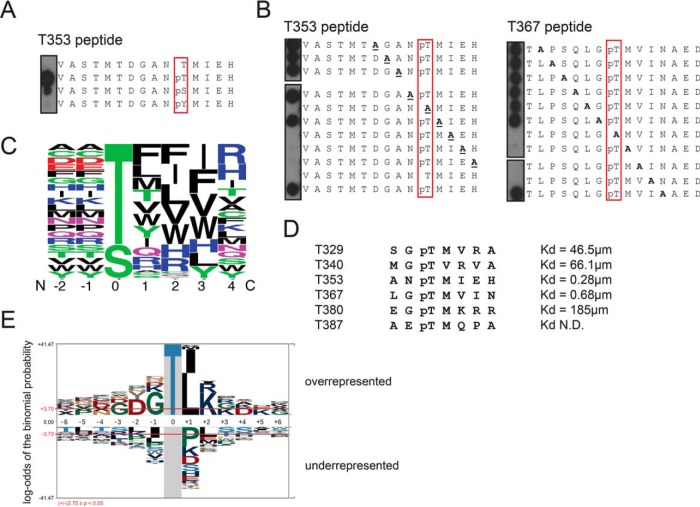
**MST1 and MST2 kinases may self-potentiate phospho-dependent binding to full-length MOB1.** *A,* Far-Western peptide array assays to define preferences for the phosphorylated amino acid at the P0 position of a MST1 T353 peptide. *B,* Positional alanine scanning of MST1 pT353 and pT367 peptides to define positions important for GST-MOB1B binding by Far-Western peptide array. *C,* Sequence logo representation of the phosphopeptide binding consensus of MOB1B determined by MST1 peptide arrays (See supplemental Fig. S2 and S3). *D,* Amino acid sequence and binding affinity of MST1 phosphopeptides for MOB1A (see Fig. 2*A* and supplemental Fig. S4*A* and S4*B*). *E,* Sequence logo representation of the substrate phosphorylation consensus for recombinant MST1 and MST2 determined from a whole-cell lysate kinase assay by SILAC labeling and quantitative phosphoproteomics (See supplemental Table S2).

We sought to explore the specificity determinants for phospho-dependent binding of MOB1B using peptide arrays and Far-Western detection, by substituting each residue individually in the human pT353 and pT367 peptide sequences. Our initial analysis revealed a strong preference for phosphothreonine over phosphoserine, and near complete loss of binding for phosphotyrosine at the P0 peptide position (Fig. 3*A*). Alanine substitutions across the pT353 and pT367 peptide sequences identified further strong binding determinants C-terminal to the phosphorylation site, whereas positions N-terminal to the phosphorylation site appeared permissive to alanine (Fig. 3*B*). To define a consensus for the preferred binding motif, we substituted positions −2 to +2 of the pT353 peptide (supplemental Fig. S2) and positions +1 to +4 of the pT367 peptide (supplemental Fig. S3) with all possible naturally occurring amino acids. A summary of the binding consensus derived from the Far-Western analysis is shown in sequence logo form in Fig. 3*C*. Most notably, in addition to the strong preference for phosphothreonine at the P0 position, a strong preference for hydrophobic residues at positions +1 to +3 was also observed. Beyond these core positions, little discrimination of sequence was detected.

As predicted based on the consensus derived from our peptide arrays, free fluorescently-labeled peptides corresponding to four other phosphothreonine-containing sequences in the linker region of MST1 (T329, T340, T380, and T387) bound much more weakly to recombinant MOB1A (Fig. 3*D*, Fig. 2*A*, supplemental Fig. S4*A*, S4*B*). The weaker binding affinities for these phosphopeptides relative to the high affinity pT353 and pT367 peptides could be rationalized by mismatches to one or more positions in the consensus (Fig. 3*C*, 3*D*). Consistent with their P0 positional binding preferences, human MOB1 bound more tightly to the MST1 pT367 peptide than to the optimized Nud1-like phosphoserine containing peptide than did yeast Mob1, and *vice versa* (Supplemental Fig. S4*C*, S4*D*,).

Interestingly, the MST1 peptide sequences that interacted most tightly with MOB1B in our peptide arrays bore sequence similarity to the phosphorylation consensus we determined for the MST1 and MST2 kinases using a whole cell lysate system (Fig. 3*E*); see Experimental Procedures and (32). Briefly, to determine the phosphorylation consensus sequence, HeLa cell were grown in SILAC (Stable isotope labeling using amino acids in cell culture) media (light, medium, heavy, see ref 33). Cell lysates were prepared with endogenous kinases inactivated using 5′-(4-fluorosulfonylbenzoyl) adenosine (FSBA), followed by buffer exchange to remove residual FSBA. Recombinantly-expressed MST1 and MST2 kinase domains were incubated with medium and heavy SILAC lysates, whereas the light SILAC lysate was used for the negative (no kinase added) control (see Experimental Procedures for details and information on label swaps). Samples were pooled, proteolyzed and phosphopeptides were enriched using titanium dioxide. Phospho-enriched samples were analyzed by quantitative mass spectrometry and phosphopeptides that were enriched in either the MST1 or MST2 samples (in comparison to the no kinase samples) were identified. Our analysis identified 619 MST1 and MST2 target phosphorylation sites (supplemental Table S2). Enriched sequences showed a strong preference for phosphothreonine over phosphoserine at the P0 position. In addition, within the phosphothreonine-containing peptides a preference for hydrophobic residues in the *p* + 1 position was observed, like the MOB1 phosphopeptide-binding consensus. Only a small degree of mismatch was observed between the MOB1B binding consensus and MST1 and MST2 kinase consensus at the *p* + 2 position, where lysine was preferred by the kinase but not selected for by MOB1B (Fig. 3E). Based on these results, we predicted that MST1 has a strong intrinsic ability to auto-phosphorylate at the T353 and T367 positions, which would create ideal binding sites for MOB1.

##### Structural Basis for High Affinity Binding of MOB1A to Optimal MST1 Phosphopeptides

**Fig. 4.**
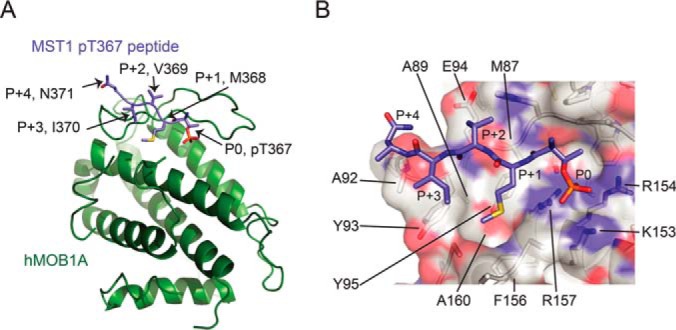
**Crystal structure of full-length MOB1A bound to a MST1 pT367 phosphopeptide.** *A,* Overview of the crystal structure of an MST1 pT367 peptide (light blue) bound to MOB1A (green). *B,* Detailed surface view of the contact region between an MST1 pT367 peptide and MOB1A. The MST1 pT367 peptide is shown in stick representation and MOB1A is shown in a transparent surface representation.

To determine the structural basis for the binding specificity of MOB1A for preferred phosphopeptide sequences, we solved the X-ray crystal structures of full-length nonphosphorylated human MOB1A bound to human MST1 pT353 and pT367 phosphopeptides at 2.5Å and 2.1Å resolution, respectively (Fig. 4*A*, 4*B*, supplemental Fig. S6*A*; see also supplemental Fig. S5 for a structure based sequence alignment and supplemental Table S3 for data collection and model refinement statistics). The observed binding modes revealed by these structures are in close agreement with the binding modes reported previously for a yeast Nud1 derived phosphopeptide in complex with human MOB1A, as well as a MST2 T378 phosphopeptide in complex with human MOB1B (supplemental Fig. S6*B*, S6*C*; see ref 21, 22). However, we note that the phosphopeptides crystallized in those earlier studies deviated to some extent from the preferred MOB1B phospho-binding consensus presented here (Fig. 3*C* and supplemental Fig. S6*B*, S6*C*).

As predicted by prior mutational data, the MST1 pT353 and pT367 phosphopeptides bound to the MOB1A phospho-binding pocket first identified by Rock *et al.* (22), with the phosphate of the P0 threonine directly coordinated by K153, R154 and R157. The strong preference for Thr over Tyr can be rationalized by the limited size of the phospho-binding pocket that cannot accommodate the bulky Tyr side chain. The preference for Thr over Ser was less obvious because the γ-methyl orients out to solvent. However, we hypothesize that the more constrained rotational mobility of the Thr *versus* Ser side chain would help to orient the attached phosphate group for optimal interactions with MOB1A (46).

The *p* + 1 side chains of M354 and M368 on MST1-pT353 and MST1-pT367 peptides respectively resided in an expansive hydrophobic surface composed by A89, A92, Y93, Y95, F156 and A160 side chains on MOB1A. The *p* + 3 side chain of I370 in the MST1-pT367 peptide oriented toward the same hydrophobic surface. The *p* + 2 side chains of I355 and V369 in MST1-pT353 and MST1-pT367 peptides respectively pointed to a distinct surface composed by M87, Y95 and the aliphatic portion of the E94 side chain on MOB1A. Thus, the hydrophobic nature of the *p* + 1 to *p* + 3 binding surfaces explained the consensus preferences for hydrophobic residues at these positions. The main chain regions beyond P-1 and *p* + 4 oriented toward solvent, which explained the lack of specificity beyond these positions.

Ni *et al.* recently reported the crystal structure of MOB1 bound to an extended phosphopeptide derived from MST2 encompassing T378 (supplemental Fig. S6*C*, ref 21). This considerably longer peptide sequence (31 residues *versus* the 15-residue peptides described here) appeared suboptimal in having disfavored basic residues at positions *p* + 2 and *p* + 3, and yet was reported to bind more tightly (120 nm) to MOB1B (21) than did the consensus matching shorter MST1 pT353 peptide. We reason that the suboptimal match to the consensus was compensated for by the observed secondary contacts involving residues far C-terminal to the phosphosite in MST2 (corresponding to an α-helical stretch of residues 391 to 397), which engaged a surface on MOB1B remote from the MOB1B phosphopeptide binding pocket. In support of this hypothesis, we observed that the orthologous peptide region in MST1 (residues 391 to 405, containing the nonphosphorylated PSFLEYF sequence) was sufficient to bind to MOB1B in our peptide array assays (supplemental Fig. S1*C*).

**Fig. 5.**
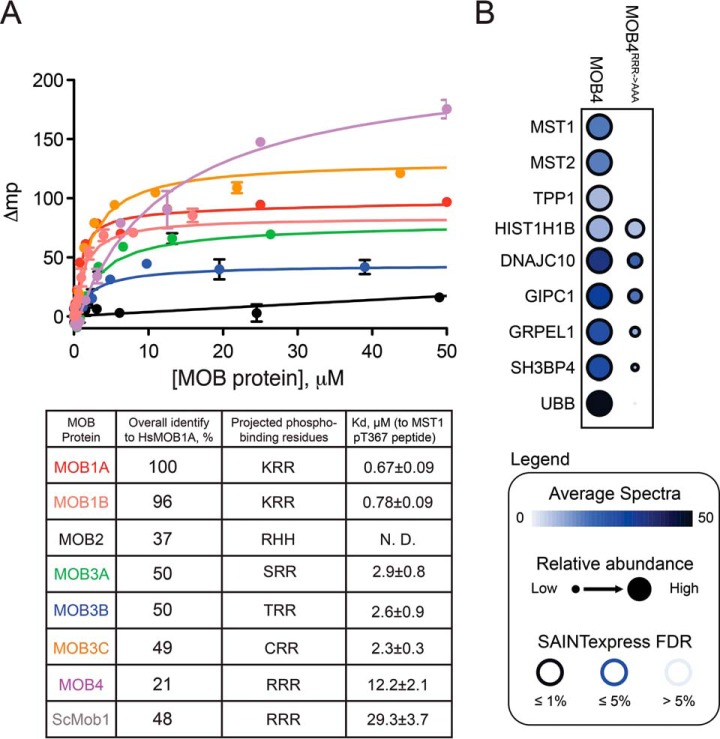
**Phospho-recognition by human MOB family proteins.** *A,* Fluorescence polarization analysis of human MOB proteins binding to a MST1 pT367 phosphopeptide (Top); Summary table of the calculated binding affinities exhibited by human MOB proteins to a MST1 pT367 phosphopeptide, along with overall sequence identity to MOB1A and the projected phosphate coordinating residues (Bottom). Data is plotted as the mean ± S.E. (*n* = 3). *B,* Protein interactors of GST-MOB4 and GST-MOB4^RRR→AAA^ from HeLa cell lysate treated with okadaic acid. The circle color maps to the averaged spectral counts detected for the prey (rows) in the purification of the baits (columns); the relative abundance across the baits for the same prey is depicted by the node size whereas the color edge maps to the confidence in identification from SAINTexpress, expressed as FDR (visualization at ProHits-viz.lunenfeld.ca). All interactions are listed in supplemental Table S4. Also see ProHits-web.lunenfeld.ca (project: “phospho-dependent Hippo interactions”).

With the exception of MOB2, all human MOB proteins display some conservation of the basic residues at positions corresponding to K153, R154 and R157 in MOB1A, suggesting that they too may possess an ability to bind phosphopeptide sequences (supplemental Fig. S5). In support of this hypothesis, we found that all human MOB proteins with the exception of MOB2 displayed quantifiable affinity for the MST1 pT367 peptide in fluorescence polarization assays, with Kds ranging from 2–3 μm for the three MOB3 proteins to 12.2 μm for MOB4 (Fig. 5*A*). To validate whether this binding was dependent of the projected phosphopeptide binding infrastructure of each MOB protein and whether it could occur in the competitive environment of a cellular lysate, we performed GST pull-down experiments with the most divergent member of the family, MOB4, which showed only modest (12.2 μm) phosphopeptide binding function *in vitro*. To this end, recombinantly expressed GST-MOB4 wild-type protein as well as a GST-MOB4 mutant in which the predicted phosphate coordinating residues R161, R162, and R165 were mutated to alanine, were employed in GST pull-down analyses using cellular lysates treated with okadaic acid. Analysis by mass spectrometry (against negative controls consisting of okadaic acid-treated lysate incubated with glutathione resin alone), recovered only a few high confidence interactors for GST-MOB4 that strikingly included both MST1 and MST2. The association with MST1 and MST2 (together with additional interactors) was dependent on the predicted phosphate coordinating residues R161, R162, and R165 (Fig. 5*B*). These results confirm that most human MOB proteins have the potential to associate with phosphorylated MST1 and 2, and that they would do so by using the same infrastructure first uncovered by Rock *et al.* (22).

##### Validation of Binding Determinants Between MST1 and MOB1 in Cells

**Fig. 6.**
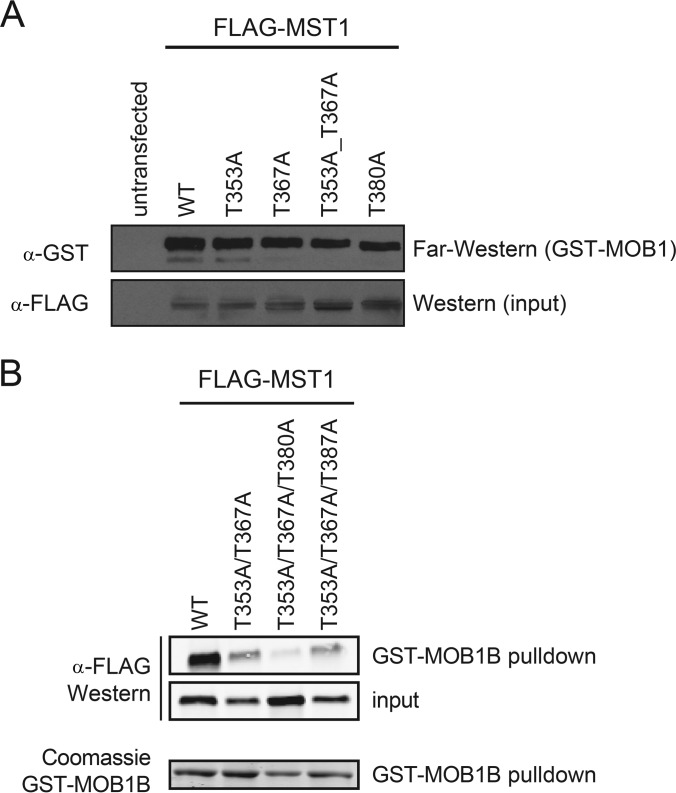
**Multiplicity of phosphosites in the MST1 interdomain linker region provides redundancy in binding to MOB1.** *A,* FLAG-tagged MST1 mutants expressed in HEK293 cells treated with okadaic acid were subjected to Far-Western analysis. *Top panel*: Far-Western blot using GST-MOB1B as a probe; *bottom panel*: anti-FLAG Western blot to verify equal loading of MST1 across mutants. *B,* GST pull down analysis of MOB1B interaction with MST1. WT and the indicated mutant versions of FLAG-MST1 were expressed in HEK293 cells treated with okadaic acid, and subjected to GST pull-down analysis with GST-MOB1B. An anti-FLAG Western blot was used to determine the recovery of FLAG-MST1 proteins in the GST-MOB1B pull-down and in the lysate (input). Loading of the GST-MOB1B protein was assessed by Coomassie staining of the SDS-PAGE gel.

Next, we sought to confirm the existence of multiple phosphorylation sites on MST1 that can independently engage with MOB1 using a cell-based system. Mutation of the two favored *in vitro* sites identified here, T353 or T367, to alanine in isolation or combined were insufficient to abrogate interaction with GST-MOB1B in a Far-Western assay (Fig. 6*A*). Similarly, a single point mutation substituting T380 (paralogous to the previously reported T378 in MST2) to alanine did not abolish MOB1B binding either (Fig. 6*A*). Given that all three phosphopeptides can each independently associate with MOB1 *in vitro*, we next constructed FLAG-tagged double and triple mutants at these sites in MST1, transfected each in HEK293 cells, stimulated the cells with okadaic acid to maximize phosphorylation-dependent binding, and prepared cell lysates. We next performed a GST pull-down analysis using GST-MOB1B with each lysate and detected interactions with MST1 by anti-FLAG immunoblotting. Although the wild-type MST1 protein readily interacted with GST-MOB1B in this assay, the MST1 mutant in which all three identified binding sites (T353, T367, and T380) were mutated to alanine displayed a marked reduction in association with MOB1B, despite the fact that it was expressed at a comparable level to the wild-type protein (Fig. 6*B*; we note that the double MST1 mutant at T353 and T367 and a triple mutant at T353, T367, and T387 also displayed a decrease in binding in comparison to wild-type MST1, though not as strikingly as the T353, T367, T380 triple mutant, especially when considering relative levels of expression). Together, these mutational studies suggest that, in a cellular context, a redundancy of sites mediate the association of MOB1 with phosphorylated MST1, and that even phosphorylation sites in a suboptimal context (such as the T380 site) can contribute to phosphorylation-dependent binding if they act in concert with secondary peptide sequences that engage an independent peptide recognition surface on MOB1 (Fig. 6*B*).

## DISCUSSION

In this study, we investigated the mechanisms behind the recruitment of MST1 and MST2 kinases to MOB1, which facilitate signal transmission in the Hippo pathway by bringing the MST1 and MST2 kinases in close vicinity to their substrates, the LATS family kinases. Activation of MST1 and MST2 kinases by upstream signals would facilitate autophosphorylation, including on sites in their linker regions (as first reported in ref 44). Here, we show that the substrate phosphorylation consensus for MST1 and MST2 kinases is compatible with the MOB1 phosphopeptide binding consensus, suggesting that auto-phosphorylation of MST1 and MST2 would create docking sites that are efficiently recognized by the phosphopeptide binding infrastructure of MOB1.

The cocrystal structures of MOB1A and MST1 pT353 and pT367 phosphopeptides reported here are in full agreement with the binding mode first reported by Rock *et al.* (22), and subsequently reported by Ni *et al.* (21), in which the basic residues, K153, R154, and R157 coordinate the phosphorylated residue. Furthermore, our peptide array experiments not only identified multiple peptide sites on MST1 that can interact with MOB1B in a phosphorylation-dependent manner, they also revealed a hydrophobic stretch of residues that can interact with MOB1B in a phosphorylation independent manner. In corroboration of the findings of Ni *et al.* (21), these peptides coincide with the nonphosphorylated sequence element that contributes to a bipartite phosphopeptide binding mechanism.

The binding features of MOB1 described above create multifold redundancy to the phosphorylation-dependent binding of MOB1 to MST1 in cells, whereby even weaker phosphopeptide binding sites can contribute. This behavior raises an important question of purpose. One possibility is that the redundancy of phosphosites in MST1 (and MST2) provides a safeguard to the mutation of a single essential site, which would be more likely to short circuit Hippo pathway function and hence disable the brake on YAP1 and TEAD oncogenic functions. A second possibility is that multiple phosphorylation sites in MST1 and MST2 allow multiple MOB1 proteins to bind each MST1 (or MST2) kinase concurrently (similar to the phenomenon in polyribosomes (47)), which could serve an amplifying role in potentiating Hippo pathway signaling to the downstream LATS1 and LATS2 kinases. A third possibility is that the presence of multiple sites, each with varied binding potential to MOB1, affords the cell an opportunity to fine tune the intensity of signaling through the Hippo pathway. The newly elucidated rules that govern the strength of phosphopeptide recognition by MOB1 empower future studies to address these questions of function in a rational and systematic manner.

Lastly, our studies support the notion that the phosphopeptide binding function of MOB1 is broadly relevant to the function of other MOB proteins. We showed that, of the seven human MOB proteins, all but one display conservation of basic residues at the projected phospho-coordinating positions and that this correlates with a measurable ability to bind pThr peptides *in vitro*. In addition, our studies show that MOB4 can interact with MST1 and MST2 in a manner dependent on its predicted phospho-coordinating infrastructure. This not only suggests that the other more similar MOB proteins will engage phosphopeptides in a physiological setting, but that they may play hitherto unanticipated roles in Hippo pathway signaling. Further characterization of the phosphopeptide binding ability and specificity of each MOB family member may assist in understanding the specific signaling functions that each carry out in a cellular context.

## DATA AVAILABILITY

X-ray co-structures have been deposited to the Protein Data Bank housed at the Center for Integrative Proteomics Research at Rutgers, The State University of New Jersey (http://www.rcsb.org/pdb/home/home.do). PDB codes 5TWH and 5TWG, for MOB1A+pT353 and MOB1A+pT367, respectively. Raw mass spectrometry files are deposited in the MassIVE repository housed at the Center for Computational Mass Spectrometry at University of California, San Diego (UCSD) (http://massive.ucsd.edu). The files associated with the determination of the MST1 and MST2 consensus have been assigned the MassIVE ID MSV000080315, ftp://massive.ucsd.edu/MSV000080315. Raw mass spectrometry files, associated peak list and results files associated with the determination of MOB4 interactors by GST pull-down assays have been deposited in ProteomeXchange through partner MassIVE as a complete submission and assigned the MassIVE ID MSV000080566 and PXD005963, ftp://massive.ucsd.edu/MSV000080566.

## Supplementary Material

Supplemental Data

## Footnotes

^1^ The abbreviations used are:

MST1: 2, Mammalian STE20-like protein kinases 1 and 2, gene names STK4 and STK3
AP-MS: Affinity purification coupled to mass spectrometry
GST: Glutathione S-transferase
HM: Hydrophobic motif
LATS1: 2, Large tumour suppressor kinases 1 and 2
MOB1A: B, Mps one binder kinase activator, 1A, 1B
OA: Okadaic acid
SDS-PAGE: Sodium dodecyl sulfate polyacrylamide gel electrophoresis
WWTR1: WW domain containing transcription regulator protein 1/protein known as TAZ
TAZ: Transcriptional coactivator with PDZ-binding domain/gene name WWTR1
YAP1: Yes associated protein 1
SAV1: Protein Salvador homolog 1
NDR1: 2, Nuclear Dbf2 related kinases 1 and 2/gene names STK38 and STK38L.

## References

[1] Harvey K. F., Pfleger C. M., and Hariharan I. K. (2003) The Drosophila Mst ortholog, hippo, restricts growth and cell proliferation and promotes apoptosis. Cell 114, 457–467 12941274 10.1016/s0092-8674(03)00557-9

[2] Jia J., Zhang W., Wang B., Trinko R., and Jiang J. (2003) The Drosophila Ste20 family kinase dMST functions as a tumor suppressor by restricting cell proliferation and promoting apoptosis. Genes Dev. 17, 2514–2519 14561774 10.1101/gad.1134003 PMC218145

[3] Justice R. W., Zilian O., Woods D. F., Noll M., and Bryant P. J. (1995) The Drosophila tumor suppressor gene warts encodes a homolog of human myotonic dystrophy kinase and is required for the control of cell shape and proliferation. Genes Dev. 9, 534–546 7698644 10.1101/gad.9.5.534

[4] Pantalacci S., Tapon N., and Leopold P. (2003) The Salvador partner Hippo promotes apoptosis and cell-cycle exit in Drosophila. Nat. Cell Biol. 5, 921–927 14502295 10.1038/ncb1051

[5] Udan R. S., Kango-Singh M., Nolo R., Tao C., and Halder G. (2003) Hippo promotes proliferation arrest and apoptosis in the Salvador/Warts pathway. Nat. Cell Biol. 5, 914–920 14502294 10.1038/ncb1050

[6] Wu S., Huang J., Dong J., and Pan D. (2003) hippo encodes a Ste-20 family protein kinase that restricts cell proliferation and promotes apoptosis in conjunction with salvador and warts. Cell 114, 445–456 12941273 10.1016/s0092-8674(03)00549-x

[7] Xu T., Wang W., Zhang S., Stewart R. A., and Yu W. (1995) Identifying tumor suppressors in genetic mosaics: the Drosophila lats gene encodes a putative protein kinase. Development 121, 1053–1063 7743921 10.1242/dev.121.4.1053

[8] Harvey K. F., Zhang X., and Thomas D. M. (2013) The Hippo pathway and human cancer. Nat. Rev. Cancer 13, 246–257 23467301 10.1038/nrc3458

[9] Johnson R., and Halder G. (2014) The two faces of Hippo: targeting the Hippo pathway for regenerative medicine and cancer treatment. Nat. Rev. Drug Discov. 13, 63–79 24336504 10.1038/nrd4161 PMC4167640

[10] Tate G., Kishimoto K., and Mitsuya T. (2015) Biallelic alterations of the large tumor suppressor 1 (LATS1) gene in infiltrative, but not superficial, basal cell carcinomas in a Japanese patient with nevoid basal cell carcinoma syndrome. Med. Mol. Morphol. 48, 177–182 25119020 10.1007/s00795-014-0086-8

[11] Tapon N., Harvey K. F., Bell D. W., Wahrer D. C., Schiripo T. A., Haber D., and Hariharan I. K. (2002) salvador Promotes both cell cycle exit and apoptosis in Drosophila and is mutated in human cancer cell lines. Cell 110, 467–478 12202036 10.1016/s0092-8674(02)00824-3

[12] Lai Z. C., Wei X., Shimizu T., Ramos E., Rohrbaugh M., Nikolaidis N., Ho L. L., and Li Y. (2005) Control of cell proliferation and apoptosis by mob as tumor suppressor, mats. Cell 120, 675–685 15766530 10.1016/j.cell.2004.12.036

[13] Chan E. H., Nousiainen M., Chalamalasetty R. B., Schafer A., Nigg E. A., and Sillje H. H. (2005) The Ste20-like kinase Mst2 activates the human large tumor suppressor kinase Lats1. Oncogene 24, 2076–2086 15688006 10.1038/sj.onc.1208445

[14] Huang J., Wu S., Barrera J., Matthews K., and Pan D. (2005) The Hippo signaling pathway coordinately regulates cell proliferation and apoptosis by inactivating Yorkie, the Drosophila Homolog of YAP. Cell 122, 421–434 16096061 10.1016/j.cell.2005.06.007

[15] Bichsel S. J., Tamaskovic R., Stegert M. R., and Hemmings B. A. (2004) Mechanism of activation of NDR (nuclear Dbf2-related) protein kinase by the hMOB1 protein. J. Biol. Chem. 279, 35228–35235 15197186 10.1074/jbc.M404542200

[16] Stegert M. R., Tamaskovic R., Bichsel S. J., Hergovich A., and Hemmings B. A. (2004) Regulation of NDR2 protein kinase by multi-site phosphorylation and the S100B calcium-binding protein. J. Biol. Chem. 279, 23806–23812 15037617 10.1074/jbc.M402472200

[17] Hergovich A., Schmitz D., and Hemmings B. A. (2006) The human tumour suppressor LATS1 is activated by human MOB1 at the membrane. Biochem. Biophys. Res. Commun. 345, 50–58 16674920 10.1016/j.bbrc.2006.03.244

[18] Hergovich A., Kohler R. S., Schmitz D., Vichalkovski A., Cornils H., and Hemmings B. A. (2009) The MST1 and hMOB1 tumor suppressors control human centrosome duplication by regulating NDR kinase phosphorylation. Curr. Biol. 19, 1692–1702 19836237 10.1016/j.cub.2009.09.020

[19] Hoa L., Kulaberoglu Y., Gundogdu R., Cook D., Mavis M., Gomez M., Gomez V., and Hergovich A. (2016) The characterisation of LATS2 kinase regulation in Hippo-YAP signalling. Cell Signal. 28, 488–497 26898830 10.1016/j.cellsig.2016.02.012

[20] Praskova M., Xia F., and Avruch J. (2008) MOBKL1A/MOBKL1B phosphorylation by MST1 and MST2 inhibits cell proliferation. Curr. Biol. 18, 311–321 18328708 10.1016/j.cub.2008.02.006 PMC4682548

[21] Ni L., Zheng Y., Hara M., Pan D., and Luo X. (2015) Structural basis for Mob1-dependent activation of the core Mst-Lats kinase cascade in Hippo signaling. Genes Dev. 29, 1416–1431 26108669 10.1101/gad.264929.115 PMC4511216

[22] Rock J. M., Lim D., Stach L., Ogrodowicz R. W., Keck J. M., Jones M. H., Wong C. C., Yates J. R. 3rd, Winey M., Smerdon S. J., Yaffe M. B., and Amon A. (2013) Activation of the yeast Hippo pathway by phosphorylation-dependent assembly of signaling complexes. Science 340, 871–875 23579499 10.1126/science.1235822 PMC3884217

[23] Couzens A. L., Knight J. D., Kean M. J., Teo G., Weiss A., Dunham W. H., Lin Z. Y., Bagshaw R. D., Sicheri F., Pawson T., Wrana J. L., Choi H., and Gingras A. C. (2013) Protein interaction network of the mammalian Hippo pathway reveals mechanisms of kinase-phosphatase interactions. Sci. Signal. 6, rs15 24255178 10.1126/scisignal.2004712

[24] Otwinowski Z., Minor W., and et al (1997) Processing of X-ray diffraction data collected in oscillation mode. Methods Enzymol. 276, 307–326 10.1016/S0076-6879(97)76066-X 27754618

[25] Stavridi E. S., Harris K. G., Huyen Y., Bothos J., Verwoerd P. M., Stayrook S. E., Pavletich N. P., Jeffrey P. D., and Luca F. C. (2003) Crystal structure of a human Mob1 protein: toward understanding Mob-regulated cell cycle pathways. Structure 11, 1163–1170 12962634 10.1016/s0969-2126(03)00182-5

[26] Cowtan K. (2006) The Buccaneer software for automated model building. 1. Tracing protein chains. Acta Crystallogr. D Biol. Crystallogr. 62, 1002–1011 16929101 10.1107/S0907444906022116

[27] Emsley P., and Cowtan K. (2004) Coot: model-building tools for molecular graphics. Acta Crystallogr. D Biol. Crystallogr. 60, 2126–2132 15572765 10.1107/S0907444904019158

[28] Winn M. D., Murshudov G. N., and Papiz M. Z. (2003) Macromolecular TLS refinement in REFMAC at moderate resolutions. Methods Enzymol. 374, 300–321 14696379 10.1016/S0076-6879(03)74014-2

[29] Picaud S., and Filippakopoulos P. (2015) SPOTing Acetyl-Lysine Dependent Interactions. Microarrays 4, 370–388 27600229 10.3390/microarrays4030370 PMC4996381

[30] Hornbeck P. V., Zhang B., Murray B., Kornhauser J. M., Latham V., and Skrzypek E. (2015) PhosphoSitePlus, 2014: mutations, PTMs and recalibrations. Nucleic Acids Res. 43, D512–D520 25514926 10.1093/nar/gku1267 PMC4383998

[31] Kean M. J., Couzens A. L., and Gingras A. C. (2012) Mass spectrometry approaches to study mammalian kinase and phosphatase associated proteins. Methods 57, 400–408 22710030 10.1016/j.ymeth.2012.06.002

[32] Knight J. D., Tian R., Lee R. E., Wang F., Beauvais A., Zou H., Megeney L. A., Gingras A. C., Pawson T., Figeys D., and Kothary R. (2012) A novel whole-cell lysate kinase assay identifies substrates of the p38 MAPK in differentiating myoblasts. Skelet. Muscle 2, 5 22394512 10.1186/2044-5040-2-5 PMC3350448

[33] Ong S. E., Blagoev B., Kratchmarova I., Kristensen D. B., Steen H., Pandey A., and Mann M. (2002) Stable isotope labeling by amino acids in cell culture, SILAC, as a simple and accurate approach to expression proteomics. Mol. Cell. Proteomics 1, 376–386 12118079 10.1074/mcp.m200025-mcp200

[34] Cox J., and Mann M. (2008) MaxQuant enables high peptide identification rates, individualized p.p.b.-range mass accuracies and proteome-wide protein quantification. Nat. Biotechnol. 26, 1367–1372 19029910 10.1038/nbt.1511

[35] O'Shea J. P., Chou M. F., Quader S. A., Ryan J. K., Church G. M., and Schwartz D. (2013) pLogo: a probabilistic approach to visualizing sequence motifs. Nat. Methods 10, 1211–1212 24097270 10.1038/nmeth.2646

[36] Kessner D., Chambers M., Burke R., Agus D., and Mallick P. (2008) ProteoWizard: open source software for rapid proteomics tools development. Bioinformatics 24, 2534–2536 18606607 10.1093/bioinformatics/btn323 PMC2732273

[37] Shteynberg D., Deutsch E. W., Lam H., Eng J. K., Sun Z., Tasman N., Mendoza L., Moritz R. L., Aebersold R., and Nesvizhskii A. I. (2011) iProphet: multi-level integrative analysis of shotgun proteomic data improves peptide and protein identification rates and error estimates. Mol. Cell. Proteomics 10, M111 007690 10.1074/mcp.M111.007690 PMC3237071 21876204

[38] Liu G., Zhang J., Larsen B., Stark C., Breitkreutz A., Lin Z. Y., Breitkreutz B. J., Ding Y., Colwill K., Pasculescu A., Pawson T., Wrana J. L., Nesvizhskii A. I., Raught B., Tyers M., and Gingras A. C. (2010) ProHits: integrated software for mass spectrometry-based interaction proteomics. Nat. Biotechnol. 28, 1015–1017 20944583 10.1038/nbt1010-1015 PMC2957308

[39] Eng J. K., Jahan T. A., and Hoopmann M. R. (2013) Comet: an open-source MS/MS sequence database search tool. Proteomics 13, 22–24 23148064 10.1002/pmic.201200439

[40] Keller A., Nesvizhskii A. I., Kolker E., and Aebersold R. (2002) Empirical statistical model to estimate the accuracy of peptide identifications made by MS/MS and database search. Anal. Chem. 74, 5383–5392 12403597 10.1021/ac025747h

[41] Teo G., Liu G., Zhang J., Nesvizhskii A. I., Gingras A. C., and Choi H. (2014) SAINTexpress: improvements and additional features in Significance Analysis of INTeractome software. J. Proteomics 100, 37–43 24513533 10.1016/j.jprot.2013.10.023 PMC4102138

[42] Knight J. D., Liu G., Zhang J. P., Pasculescu A., Choi H., and Gingras A. C. (2015) A web-tool for visualizing quantitative protein-protein interaction data. Proteomics 15, 1432–1436 25422071 10.1002/pmic.201400429

[43] Hwang E., Ryu K. S., Paakkonen K., Guntert P., Cheong H. K., Lim D. S., Lee J. O., Jeon Y. H., and Cheong C. (2007) Structural insight into dimeric interaction of the SARAH domains from Mst1 and RASSF family proteins in the apoptosis pathway. Proc. Natl. Acad. Sci. U.S.A. 104, 9236–9241 17517604 10.1073/pnas.0610716104 PMC1890478

[44] Glantschnig H., Rodan G. A., and Reszka A. A. (2002) Mapping of MST1 kinase sites of phosphorylation. Activation and autophosphorylation. J. Biol. Chem. 277, 42987–42996 12223493 10.1074/jbc.M208538200

[45] Golemis E. (2002) Protein-protein interactions : a molecular cloning manual, Cold Spring Harbor Laboratory Press, Cold Spring Harbor, N.Y.

[46] Chen C., Ha B. H., Thevenin A. F., Lou H. J., Zhang R., Yip K. Y., Peterson J. R., Gerstein M., Kim P. M., Filippakopoulos P., Knapp S., Boggon T. J., and Turk B. E. (2014) Identification of a major determinant for serine-threonine kinase phosphoacceptor specificity. Mol. Cell 53, 140–147 24374310 10.1016/j.molcel.2013.11.013 PMC3898841

[47] Warner J. R., Knopf P. M., and Rich A. (1963) A multiple ribosomal structure in protein synthesis. Proc. Natl. Acad. Sci. U.S.A. 49, 122–129 13998950 10.1073/pnas.49.1.122 PMC300639

