# Supplementary material for: MOB1 Mediated Phospho-recognition in the Core Mammalian Hippo Pathway
Source: Mol Cell Proteomics. 2017 Apr 3;16(6):1098–110. doi: 10.1074/mcp.M116.065490 (PMC5461540; doi:10.1074/mcp.M116.065490)
Supplement: Supplemental Data [file supp_16_6_1098__index.html]

MOB1 mediated phospho-recognition in the core mammalian Hippo pathway — MOB1 Mediated Phospho-recognition in the Core Mammalian Hippo Pathway — Phospho-recognition by MOB1 — Supplemental Data 

# MOB1 Mediated Phospho-recognition in the Core Mammalian Hippo Pathway

## Supplemental Data

- Supplementary Figures (.pdf, 1.5 MB) - All Supplementary Figures for Couzens et al.
- Supplemental Table 4 (.xlsx, 119 KB) - SAINTexpress results for GST-MOB4 and GST-MOB4 3RtoA with HeLa lysate
- Supplemental Table 2 (.xlsx, 253 KB) - Peptides differentially phosphorylated by MST1 and MST2 in an in vitro assay
- Supplemental Table 3 (.doc, 49 KB) - Data collection and refinement statistics
