# Supplementary material for: MOB1 Mediated Phospho-recognition in the Core Mammalian Hippo Pathway
Source: Mol Cell Proteomics. 2017 Apr 3;16(6):1098–110. doi: 10.1074/mcp.M116.065490 (PMC5461540; doi:10.1074/mcp.M116.065490)
Supplement: Supplemental Data [file 10.1074_M116.065490_mcp.M116.065490-1.pdf]

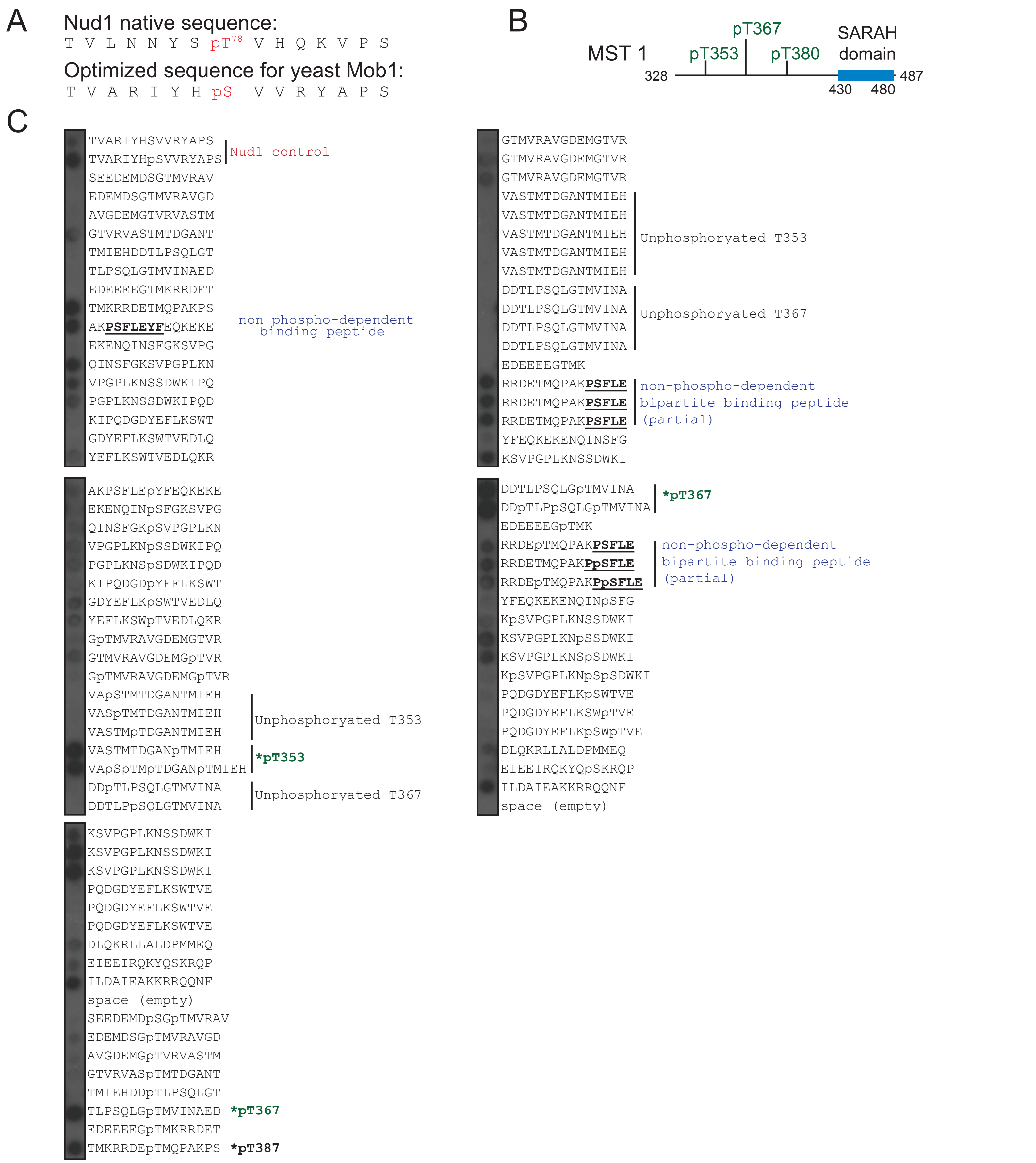


**Supplemental Fig. 1.** Binding analysis of MOB1B to peptide epitopes in the inter domain linker region of MST1. A, Sequence of (*top*) a Nud1 native phosphopeptide found to interact with yeast Mob1 or (*bottom*) a Nud1-like phosphopeptide optimized for yeast Mob1 binding that was co-crystalized with human MOB1A by Rock *et al.* (2013; *Science*, **340:**871–5). B, Schematic showing the relative positions of T353, T367, T380 and the SARAH domain in the C-terminal portion of MST1. C, 15-residue peptide spot array of MST1 probed with GST-MOB1B. Peptides containing pT353 and pT367 are indicated, as well as a non-phospho-dependent binding sequence shown previously to contribute to bipartite recognition (Ni *et al.*, 2015, *Genes Dev.*, **29:**1416–31).

## T353 peptide -2 to +2 Alanine walk

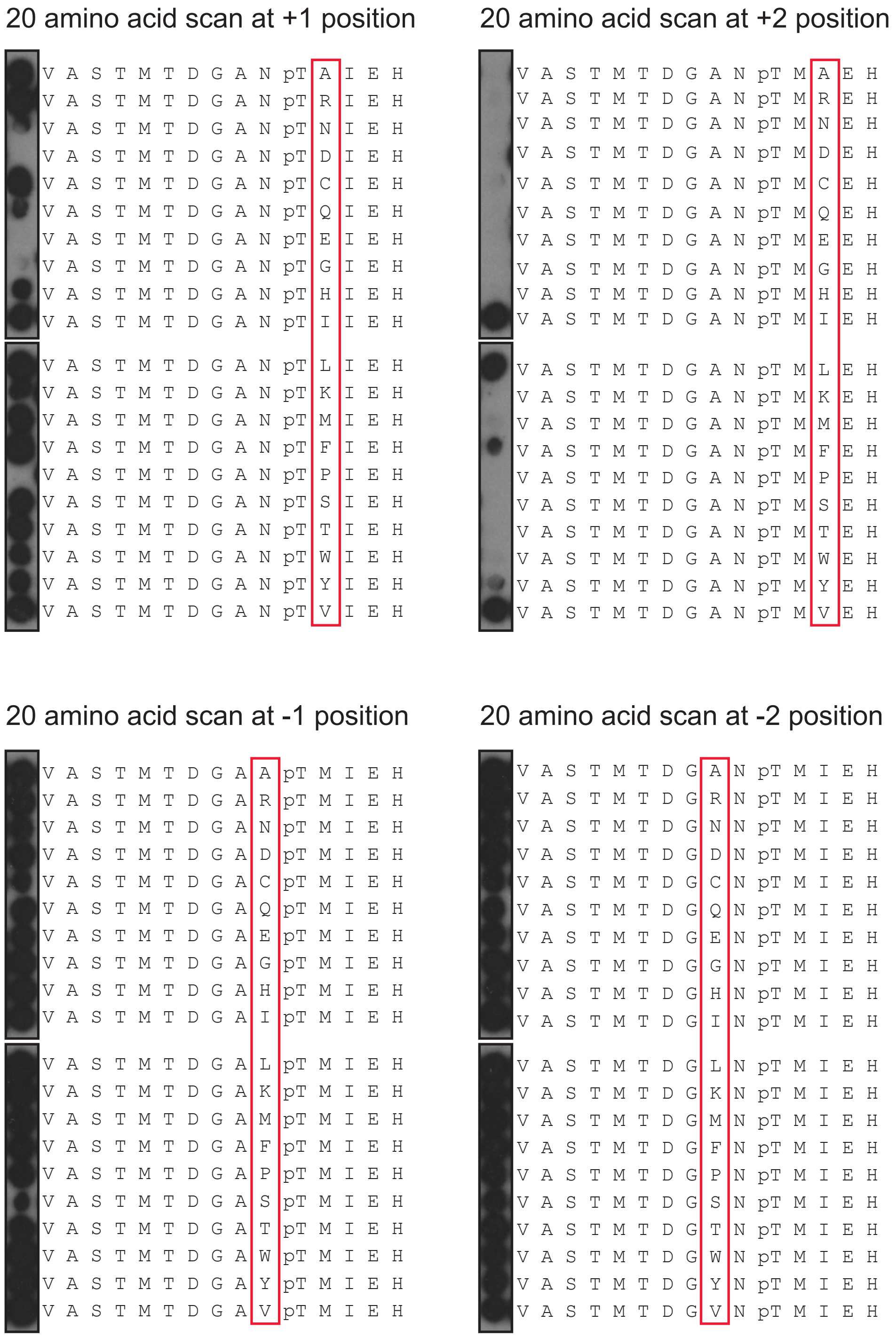


**Supplemental Fig. 2.** Binding analysis of MOB1B to single site variants of a MST1 T353 phosphopeptide. Positions P+1, P+2 P-1, and P-2 relative to the P0 pT353 site were substituted individually for each of the 20 natural occurring amino acids as indicated. Peptide spot arrays were tested for binding with GST-MOB1B in a Far-Western assay.

## T367 peptide +1 to +4 Alanine walk

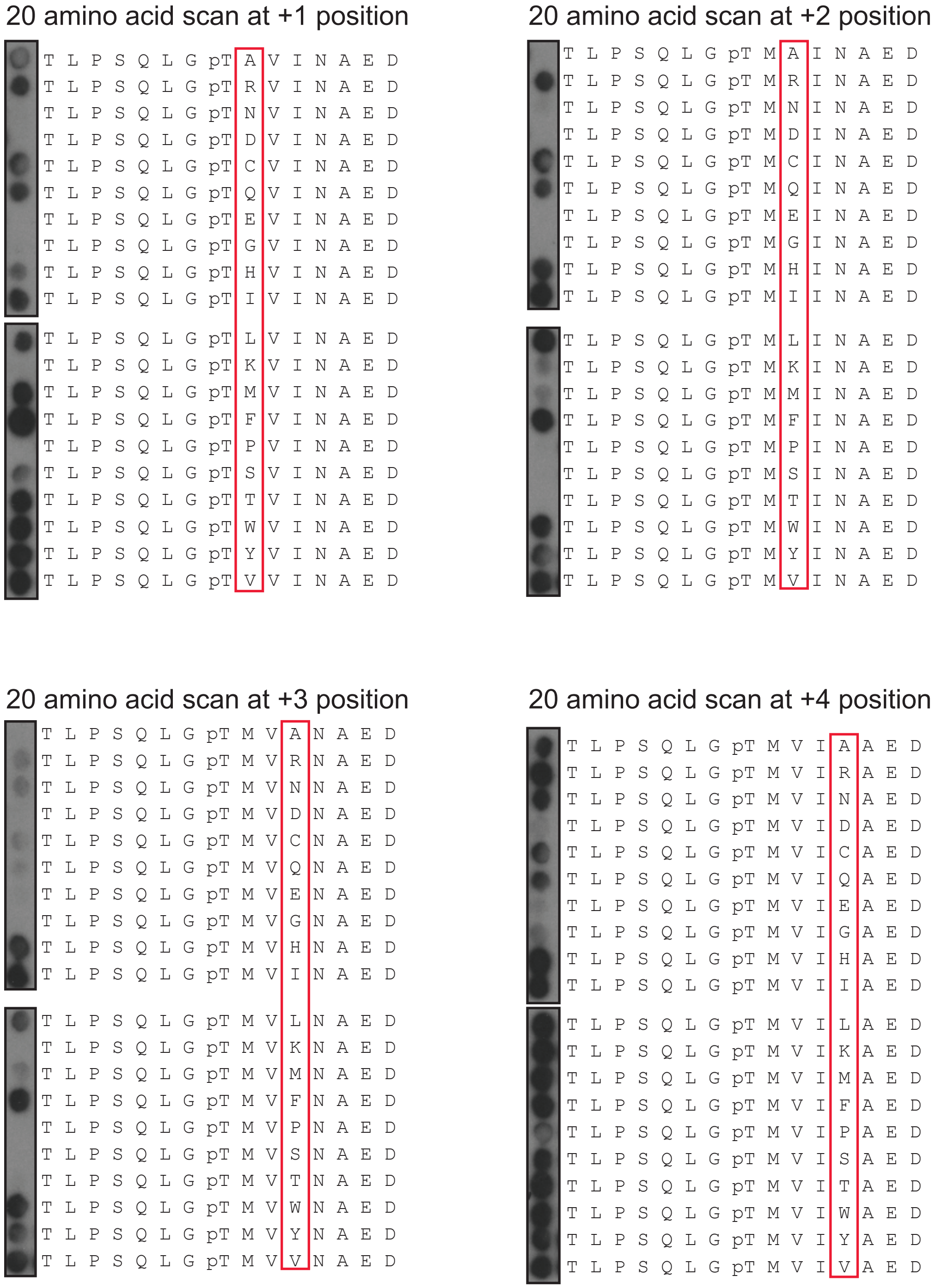


**Supplemental Fig. 3.** Binding analysis of MOB1B to single site variants of a MST1 T367 phosphopeptide. Positions P+1, P+2, P+3, and P+4 relative to the P0 pT367 site were substituted individually for each of the 20 natural occurring amino acids as indicated. Peptide spot arrays were tested for binding with GST-MOB1B in a Far-Western assay.

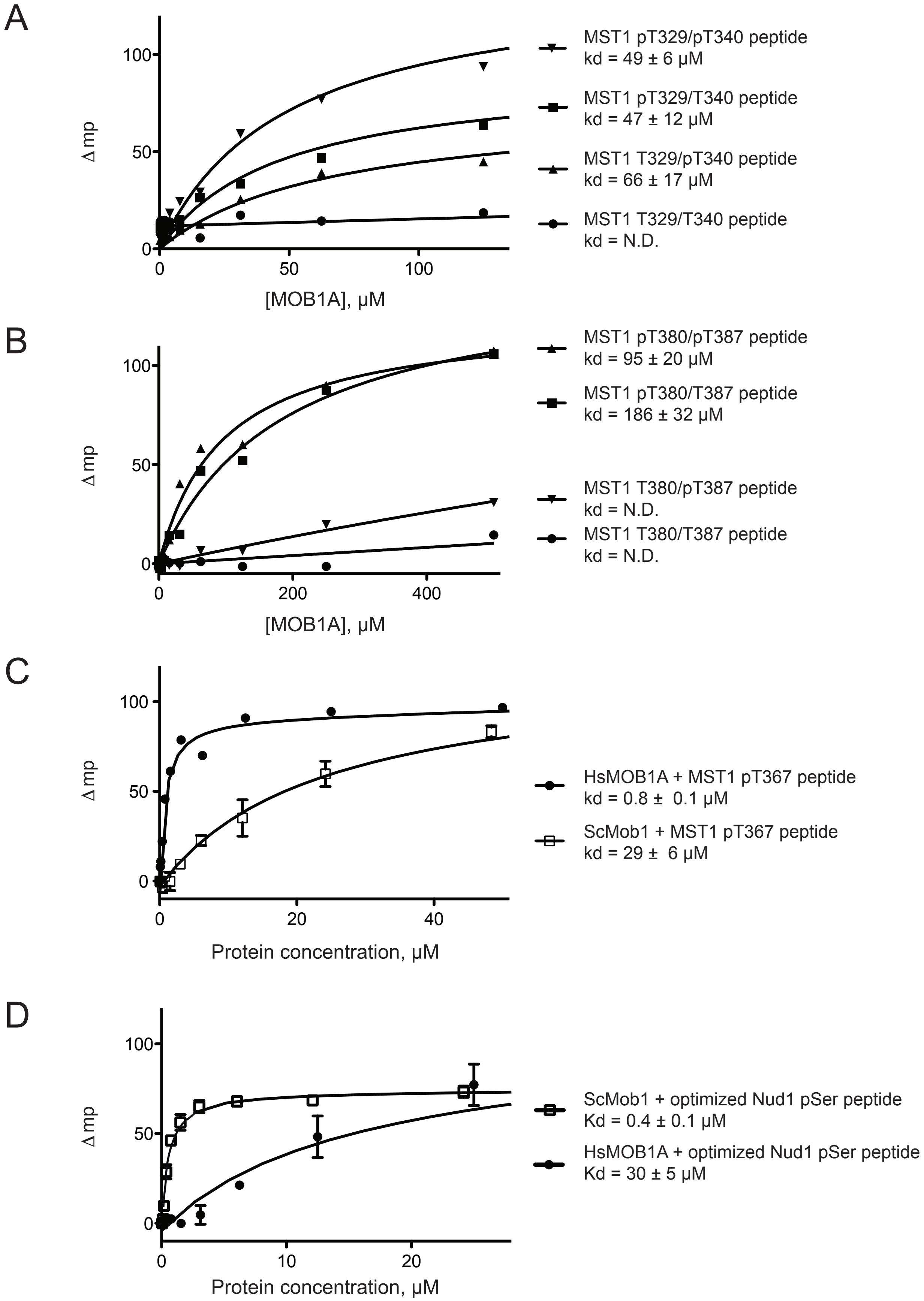


**Supplemental Fig. 4.** Binding analysis of human and yeast MOB proteins to MST1 phosphopeptides and to an optimized Nud1-like phosphopeptide. A, Fluorescence polarization analysis of MOB1A binding to differentially phosphorylated MST1 15-mer peptides containing T329 and T340. B. Fluorescence polarization analysis of MOB1A binding to differentially phosphorylated MST1 15-mer peptides containing T380 and T387. C, Fluorescence polarization analysis of human MOB1A (HsMOB1A) and yeast Mob1 (ScMob1) binding to the MST1 phosphopeptide pT367. D, Fluorescence polarization analysis of human MOB1A (HsMOB1A) and yeast MOB1 (ScMob1) binding to the optimized Nud1-like phosphoserine containing peptide. Data is plotted as the mean ± SEM (n=3).

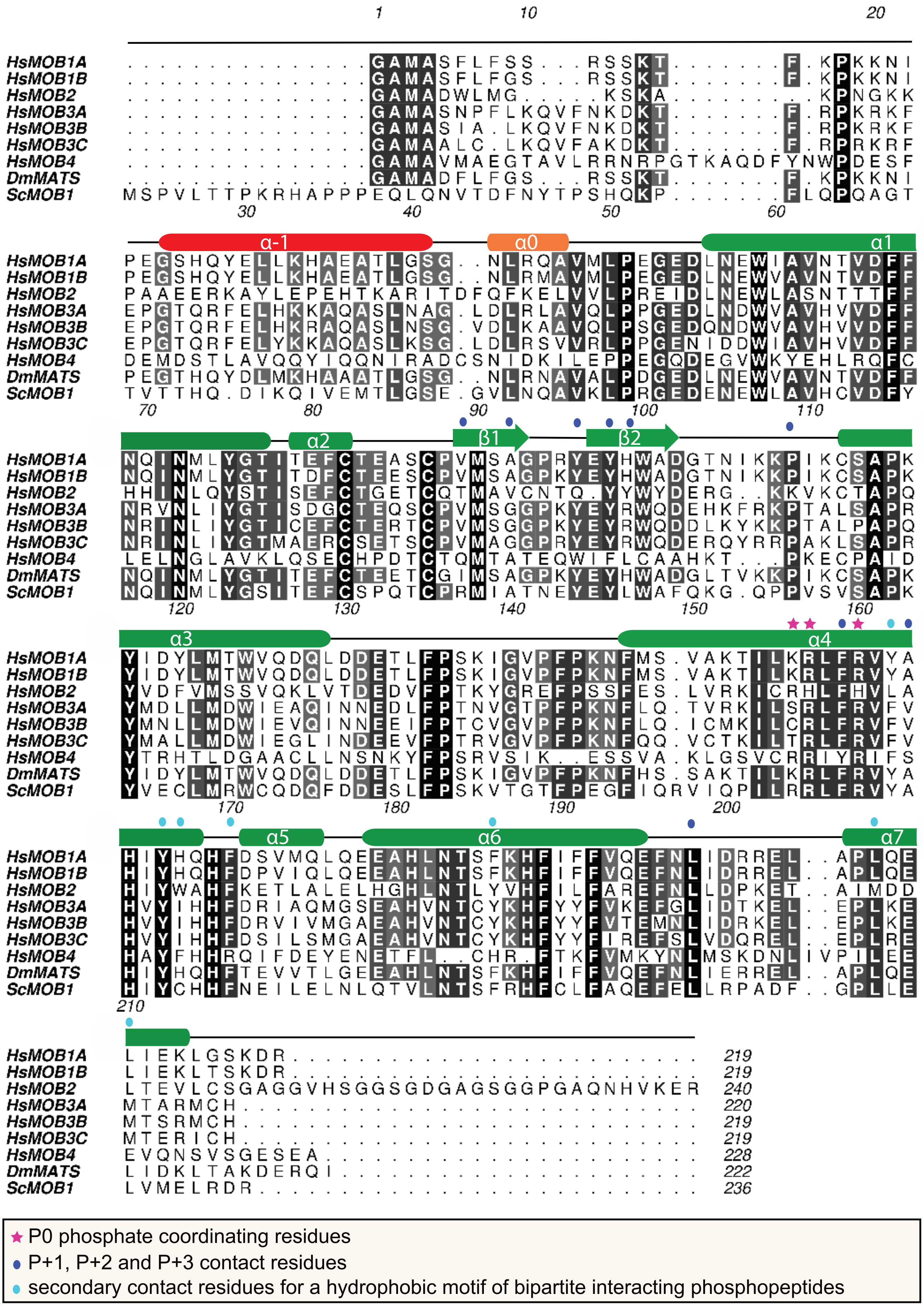


**Supplemental Fig. 5.** Structure-based sequence alignment of MOB proteins. *Homo sapiens* (*H.s.*) MOB1A (Q9H8S9-1), *H.s.* MOB1B (Q7L9L4-1), *H.s.* MOB2 (Q70IA6-1), *H.s.* MOB3A (Q96BX8-1), *H.s.* MOB3B (Q86TA1-1), *H.s.* MOB3C (Q70IA8-1), *H.s.* MOB4 (Q9Y3A3-1), Drosophila melanogaster (*D.m.*) MATS (Q95RA8-1) and *Saccharomyces cerevisiae* (*S.c.*) MOB1A (P40484-1) are shown. Contact residues are indicated as per inset.

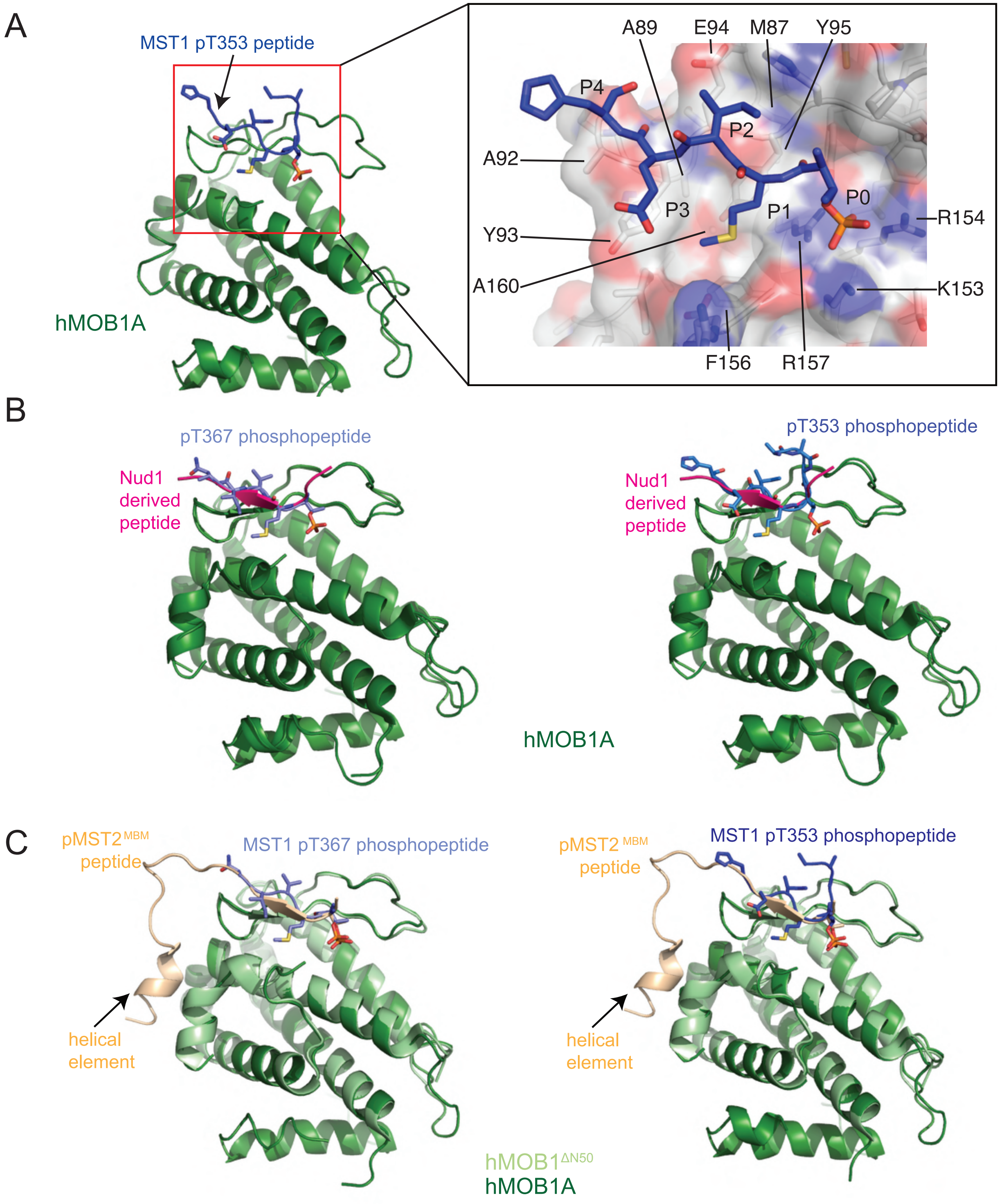


**Supplemental Fig. 6.** Structural analysis of MOB1A binding to high-affinity phosphopeptide ligands from MST1. A, Ribbons diagram of the overall structure of MOB1A bound to a pT353-containing peptide from MST1. Inset: Zoom in surface representation of the molecular interactions governing the specificity of MOB1A phosphopeptide recognition. B, Superposition of complexes of MOB1A bound to a high-affinity pT353 peptide (*right*) and a high-affinity pT367 peptide (*left*) of MST1 with a complex of MOB1A bound to a sub-optimal phosphopeptide derived from yeast Nud1 (PDB: 4JIZ; Rock *et al*., 2013; *Science*, **340:**871–5). C, Superposition of complexes of MOB1A bound to a high affinity pT353 peptide (*right*) and a high affinity pT367 peptide (*left*) of MST1 with a complex of MOB1B bound to high-affinity phosphopeptide of MST2 (orange; PDB: 5BRK; Ni *et al*., 2015, *Genes Dev*., **29:**1416–31). Note that the 31-residue MST2-MBM phosphopeptide is a suboptimal match to the MOB1B phosphopeptide binding consensus but compensates by forming additional bipartite contacts with the core domain of MOB1A through the short highlighted helical element.
