# Supplementary material for: MOB1 Mediated Phospho-recognition in the Core Mammalian Hippo Pathway
Source: Mol Cell Proteomics. 2017 Apr 3;16(6):1098–110. doi: 10.1074/mcp.M116.065490 (PMC5461540; doi:10.1074/mcp.M116.065490)
Supplement: Supplemental Data [file 10.1074_M116.065490_mcp.M116.065490-4.doc]

**Table 3** **Data collection and refinement statistics (molecular replacement)**

|  | MOB1A+pT353 | MOB1A+pT367 |
| --- | --- | --- |
| **Data collection** |  |  |
| Space group | P43212 | P43212 |
| Cell dimensions   |  |  |
| *a*, *b*, *c* (Å) | 60.986/60.986/138.486 | 60.68/60.68/137.64 |
|     *α, β, γ* (°) | 90 90 90 | 90 90 90 |
| Resolution (Å) | 2.5-27.3 | 2.1-55.6 |
| *R*_merge_, | 7.56 (59.3) | 10.8 (60.8) |
| *I/*σ(*I*) | 28.4 (5.4) | 30.1 (4.1) |
| Completeness (%) | 98.2 | 96.5 |
| Redundancy | 3.2 | 2.8 |
|  |  |  |
| **Refinement** |  |  |
|  |  |  |
| No. reflections | 9638 | 16434 |
| *R*_work_ / *R*_free_ | 22.4/25.4 | 21.4/26.1 |
| No. atoms |  |  |
| Protein | 1611 | 1665 |
| Ligand/ion (specify/describe) | 1 | 2 |
| Water | 32 | 42 |
| *B* factors |  |  |
| Protein | 60.7 | 67.5 |
| R.m.s. deviations |  |  |
| Bond lengths (Å) | 0.012 | 0.009 |
| Bond angles (°) | 1.47 | 1.241 |
|  |  |  |
|  |  |  |
|  |  |  |
